# Dynamic remodeling of lipids coincides with dengue virus replication in the midgut of *Aedes aegypti* mosquitoes

**DOI:** 10.1371/journal.ppat.1006853

**Published:** 2018-02-15

**Authors:** Nunya Chotiwan, Barbara G. Andre, Irma Sanchez-Vargas, M. Nurul Islam, Jeffrey M. Grabowski, Amber Hopf-Jannasch, Erik Gough, Ernesto Nakayasu, Carol D. Blair, John T. Belisle, Catherine A. Hill, Richard J. Kuhn, Rushika Perera

**Affiliations:** 1 Department of Microbiology, Immunology and Pathology, Colorado State University, Fort Collins, Colorado, United States of America; 2 Markey Center for Structural Biology, Department of Biological Sciences, Purdue University, West Lafayette, Indiana, United States of America; 3 Entomology Department Purdue University, West Lafayette, Indiana, United States of America; 4 Metabolite Profiling Facility (MPF), Bindley Bioscience Center, Purdue University, W. Lafayette, Indiana, United States of America; 5 Computational Life Sciences Core, Bindley Bioscience Center, Purdue University, W. Lafayette, Indiana, United States of America; 6 Purdue Institute of Inflammation, Immunology and Infectious Disease, Purdue University, West Lafayette, Indiana, United States of America; University of Pennsylvania School of Medicine, UNITED STATES

## Abstract

We describe the first comprehensive analysis of the midgut metabolome of *Aedes aegypti*, the primary mosquito vector for arboviruses such as dengue, Zika, chikungunya and yellow fever viruses. Transmission of these viruses depends on their ability to infect, replicate and disseminate from several tissues in the mosquito vector. The metabolic environments within these tissues play crucial roles in these processes. Since these viruses are enveloped, viral replication, assembly and release occur on cellular membranes primed through the manipulation of host metabolism. Interference with this virus infection-induced metabolic environment is detrimental to viral replication in human and mosquito cell culture models. Here we present the first insight into the metabolic environment induced during arbovirus replication in *Aedes aegypti*. Using high-resolution mass spectrometry, we have analyzed the temporal metabolic perturbations that occur following dengue virus infection of the midgut tissue. This is the primary site of infection and replication, preceding systemic viral dissemination and transmission. We identified metabolites that exhibited a dynamic-profile across early-, mid- and late-infection time points. We observed a marked increase in the lipid content. An increase in glycerophospholipids, sphingolipids and fatty acyls was coincident with the kinetics of viral replication. Elevation of glycerolipid levels suggested a diversion of resources during infection from energy storage to synthetic pathways. Elevated levels of acyl-carnitines were observed, signaling disruptions in mitochondrial function and possible diversion of energy production. A central hub in the sphingolipid pathway that influenced dihydroceramide to ceramide ratios was identified as critical for the virus life cycle. This study also resulted in the first reconstruction of the sphingolipid pathway in *Aedes aegypti*. Given conservation in the replication mechanisms of several flaviviruses transmitted by this vector, our results highlight biochemical choke points that could be targeted to disrupt transmission of multiple pathogens by these mosquitoes.

## Author summary

The *Aedes aegypti* mosquito transmits arboviruses that cause dengue, Zika, chikungunya and yellow fever. These viruses are endemic in tropical and subtropical regions of the world placing 2.5 billion people at risk of infection. Transmission is critically dependent upon the replication of these viruses in both human and mosquito hosts. Successful viral replication is greatly influenced by the biochemical environment of the host cell or tissue and flaviviruses rearrange this environment to benefit their needs. Host-cell derived metabolites such as lipids, sugars and amino acids are utilized to produce progeny virions, help evade the host immune system and enable successful completion of the life cycle. In this study, we applied high-resolution mass spectrometry to understand the alteration of the biochemical landscape of the mosquito during infection by dengue virus. We focused on the mosquito midgut, as this is the initial site of infection. We identified several metabolites that exhibited dynamic profiles during the course of viral infection and replication. By pinpointing biochemical “choke points” required for viral replication, we can devise strategies that will stall virus replication in the mosquito and prevent its transmission to humans.

## Introduction

The transmission cycle of dengue viruses (DENV) require a human host and mosquito vector. Mosquitoes acquire DENV via feeding on the blood of an infected human. The blood meal is deposited in the midgut of the mosquito and infection is first established in the midgut epithelium [1]. While digestion of the blood meal is complete within 48 hours [2], viral replication in the midgut tissue reaches its peak only at 7–8 days post-blood meal (pbm) ingestion [1]. Subsequently, the virus disseminates from the midgut and infects other tissues including the fat body and salivary glands. Approximately 10–14 days pbm the salivary glands become infected and the virus can be transmitted in the saliva to a human when the mosquito acquires another blood meal. Since the successful transmission of this virus depends greatly upon its ability to replicate efficiently in several mosquito tissues, the local biochemical and physical environment of each tissue plays a critical role in virus propagation.

In both human and mosquito cells, lipids play an integral role in the life cycle of DENV [3–7]. Host-derived membranes are incorporated into a lipid envelope that surrounds the capsid protein and genomic RNA of DENV particles [8]. This membranous structure facilitates virus release from infected cells by budding into the endoplasmic reticulum and re-entry into new cells through fusion of virus-host membranes [7, 9, 10]. Additionally, electron tomography has revealed that significant rearrangements of host cell membrane architecture occur upon DENV infection [4, 7, 11–13]. Virus-induced membrane structures are required as platforms for virus replication and assembly and protect replicating genomes from antiviral defense mechanisms of the host. They have been identified in DENV-infected human and mosquito cells and the midgut epithelium and salivary glands of *Culex* mosquitoes infected with West Nile virus [14]. This intracellular membrane reorganization imposes a significant metabolic cost to the host cell. It requires activation of biosynthetic processes, and the trafficking and degradation of lipids and other related molecules. Our previous studies investigated the perturbation of lipid homeostasis in C6/36 mosquito cells following infection with DENV [5]. We identified lipids involved in maintaining the stability, permeability and curvature of membranes, as well as bioactive lipid molecules that were significantly changed upon infection. Specifically, a burst of glycerophospholipids was observed coincident with viral replication kinetics. This burst of lipids was attributed to the activity of fatty acid synthase (FAS), a key enzyme in glycerophospholipid biosynthesis. Inhibition of this enzyme was detrimental for DENV replication in both human and mosquito cells [5, 6]. Collectively, these data demonstrate that lipids play critical roles in DENV infection in cell culture models.

Although lipid biochemistry has been studied in mosquitoes for several years, very little is known about the relationship between virus and mosquito host and the alteration of intracellular lipids that underpins infection capacity. In this study, we used high-resolution mass spectrometry to explore metabolic changes in the midgut of *Ae*. *aegypti* exposed to DENV-containing blood meals as this tissue represents the crucial site of initial viral replication. Using a time course study, we compared metabolic profiles of infected and uninfected midguts at early-, mid- (peak viral replication) and late time points post-infection. Our results demonstrate significant fluctuations in molecules that function as membrane building blocks, bioactive messengers, energy storage molecules and intermediates in lipid biosynthesis and lipolysis pathways. Presumably these changes represent both manipulation of cellular resources for viral replication as well as the cellular response to infection. They may result from either *de novo* biosynthesis or the consumption/conversion of metabolites. Additionally, import/export may contribute to the perturbation of metabolite pools. Import pathways are specifically important in the mosquito for molecules such as cholesterol and its derivatives since they cannot be synthesized *de novo* [15, 16]. Many unidentified metabolites (not found in currently available databases that include animal, plant, fungal and bacterial metabolites and synthetic compounds) were also significantly perturbed during virus infection and may represent mosquito-specific metabolites that have not yet been annotated. The flaviviruses transmitted by this vector share similar replication mechanisms; identifying metabolic bottlenecks that condition vector competence and transmission of these pathogens could be exploited for novel transmission-blocking interventions for control of globally important diseases.

## Results

### DENV type 2 infection of the *Ae*. *aegypti* mosquito vector

**Fig 1:**
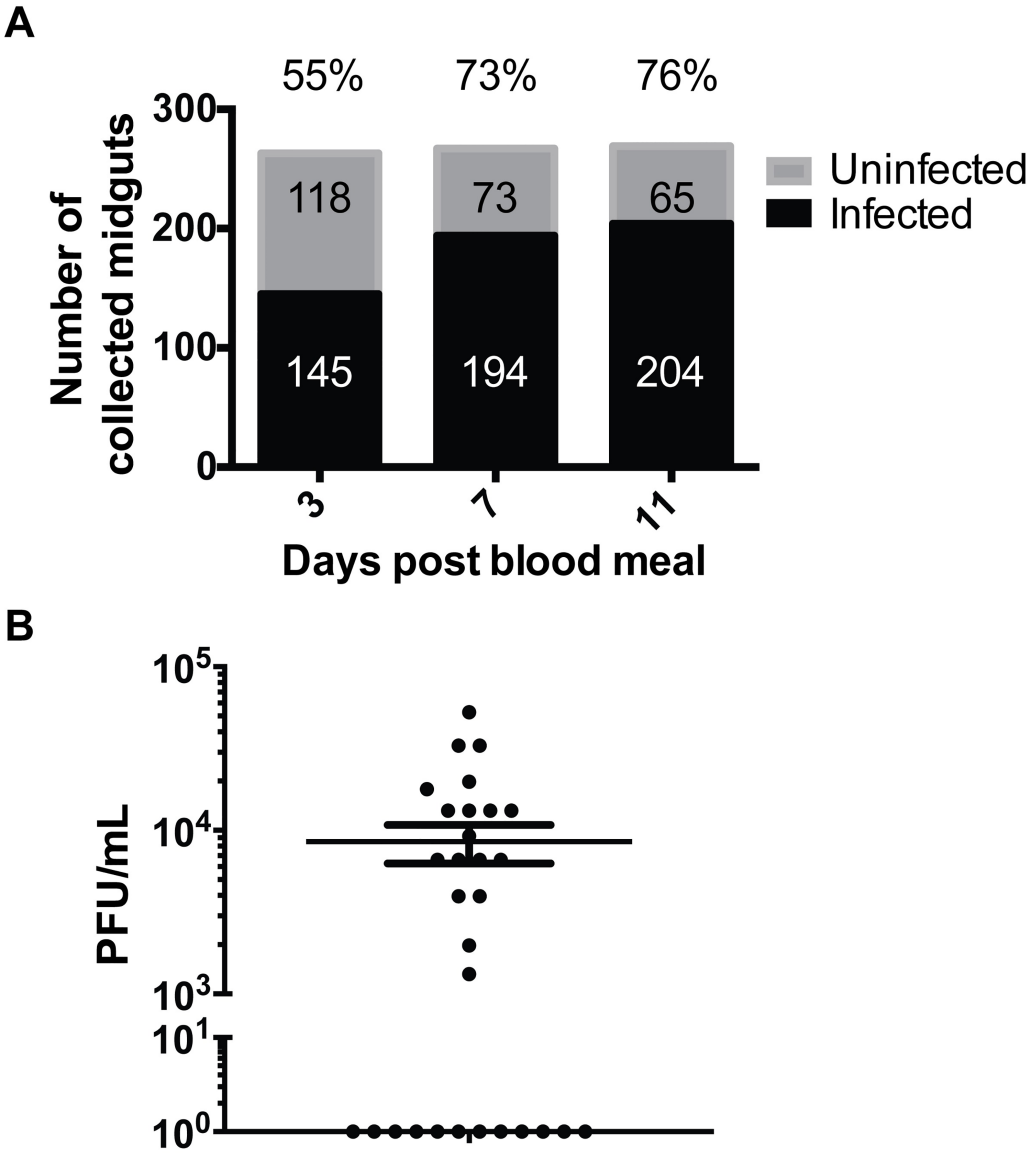
DENV infection in mosquitoes. Individual midguts dissected from DENV-fed mosquitoes (n = 799) were tested for the presence of viral RNA by qRT-PCR. (A) Percent of virus-RNA positive midguts are shown above the bar graphs. (B) Infectious viral titers of a representative subset (n = 30) of whole mosquitoes harvested on day 11 pbm were determined to evaluate the range of titers observed in the experiment following dissemination.

To profile the alterations in the metabolic environment of the mosquito midgut over time post-blood feeding and during the course of DENV infection in the mosquito vector, *Ae*. *aegypti* Chetumal strain mosquitoes were fed an infectious blood meal containing DENV type 2 strain Jamaica-1409 (JAM-1409; blood titer 1x10^7^ PFU/mL) or exposed to a noninfectious blood meal as a negative control (S1 Fig). Midguts were dissected from the mosquitoes on days 3, 7 and 11 pbm representing early viral infection in the midgut (day 3), high replication activity in the midgut with early dissemination to the salivary glands (day 7), and high replication activity in salivary glands and other tissues with virus being cleared from the midgut (day 11). Immediately after dissection, individual midguts were tested for DENV RNA by qRT-PCR (Fig 1A). To ensure that the metabolic profile of the midgut tissues accurately represented the infected environment, every individual midgut was evaluated by qRT-PCR for DENV RNA and only infected tissues were pooled. On day 3 pbm, viral genomes were detected in 55% of the dissected midguts, and on days 7 and 11 pbm, detection increased to 73% and 76% respectively. Plaque assays of whole mosquito homogenates harvested on day 11 pbm (n = 30) indicated that 60% of mosquitoes showed detectable infectious virus averaging 8.5x10^3^ PFU/mL (Fig 1B). These were similar infection rates to those reported previously by Salazar et al, 2007 [1]. Two independent pools of ~100 tissues were included for each treatment group and time point representing a total of 200 biological samples. The metabolic profiles observed in the infected tissues represent true biological deviations corresponding to the infected state. It should be noted however, that estimation of variance and statistical power are compromised by the small sample sizes since the maximum limit of samples acquirable in order to maintain metabolite integrity at a given time point is limited.

### LC-MS data analysis of mosquito midguts following DENV infection

Mosquito metabolites in both polar and non-polar phase extracts were analyzed by LC-MS in both positive and negative ionization modes to obtain the most comprehensive coverage possible. Experimental data from each extract in each mode were analyzed separately by the workflow described in materials and methods and shown in S1 Fig. Only features (molecular identities defined by a unique mass to charge ratio, *m/z* and retention time) detected in both pools (representing 200 biological replicates) were considered ‘present’ in the samples. For the midguts, a total of 6,103 molecular features from all modes, treatments and time-points pbm were detected. These features were subjected to statistical analysis to compare the differences in metabolite abundance between DENV-infected and uninfected midguts at each time point pbm. The features that showed statistically significant differences in abundance (|log_2_ fold change|) ≥ 1 and p-value < 0.05; total of 936 features) were identified using LIPID MAPS, HMDB, and Metlin databases. Approximately 39% of the significantly different features (363 molecules) were identifiable to an accuracy of < 6 parts per million (ppm) (Table 1 and S1 Table). About 93% of identified and significantly different features (341 molecules) have recognizable biological relevance. The metabolites detected in the non-polar phase from both negative and positive ionization modes comprised glycerophospholipids (GPs), sphingolipids (SPs), glycerolipids (GLs), sterols, fatty acyls, acyl-carnitines, prenols, polyketides, amino acids and peptides and other organic compounds. The metabolites detected in polar phases from both detection modes also contained the above molecular species with the exception of GLs and contained nucleosides instead. All nomenclatures and categories for lipids are based on the LIPID MAPS comprehensive classification system [17].

**Table 1:** Summary of metabolites from mosquito midguts detected across all time points and treatments.

Modes of detection	Nonpolar—Negative	Nonpolar—Positive	Polar—Negative	Polar—Positive	Total
Detected features	1,403	1,864	313	2,523	**6,103**
Features with differential abundance*	106	557	66	207	**936**
Identifiable features**	450	787	110	785	**2,132**
Identifiable features with differential abundance**	28	226	29	80	**363**

* Features that showed significant differences (|log_2_ fold change| ≥ 1 and p < 0.05) in abundance between DENV-infected and uninfected midguts

** Identification classified as parts per million (ppm) < 6

### Specific lipids levels are elevated during DENV replication in the midgut of *Ae*. *aegypti*

**Fig 2:**
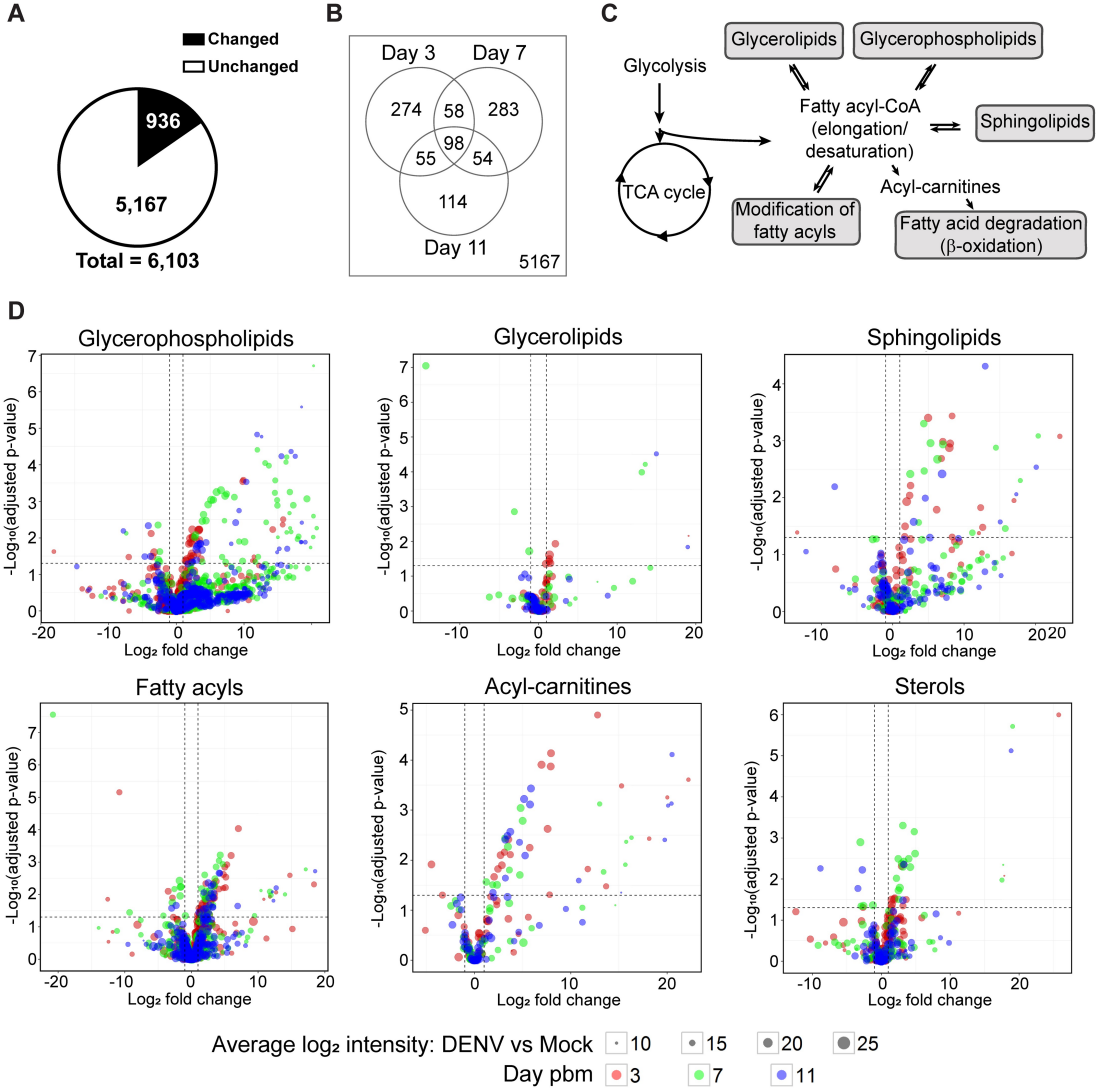
Metabolic profile of the mosquito midgut during the course of DENV infection. Significant changes were observed in the metabolic profile of the midgut upon infection. (A) The pie chart shows numbers of features in the midgut detected on days 3, 7 and 11 following a DENV-infectious blood meal compared to a noninfectious blood meal with significantly altered levels of abundance (|log_2_ fold change| ≥ 1 and p-value < 0.05) in black and nonsignificantly altered levels of abundance (|log_2_ fold change| < 1 or p-value ≥ 0.05) in white. (B) Venn diagram shows numbers of features that were altered in abundance in DENV-infected midguts compared to uninfected midguts on the days 3, 7, and 11 pbm. (C) Overview of lipid classes observed in this study and their relationship to each other within metabolic pathways. Fatty acyl-CoAs can be *de novo* synthesized from intermediates in central carbon metabolism. They can be further modified or incorporated into several classes of more complex lipids or converted to acyl-carnitine for energy production. (D) Volcano plots show the abundances of metabolites from different classes of lipids detected on days 3, 7 and 11 pbm in DENV-infected versus uninfected midguts. The vertical dashed lines indicate a 2-fold change in abundance and the horizontal dashed line indicates a p-value = 0.05.

To explore the metabolic environment in the mosquito midgut during DENV infection, we compared the metabolite repertoire of DENV-infected midguts to uninfected controls. A summary of the data is shown in Fig 2 and S1 Table. Of 6,103 features detected, 936 features (15%) have differential abundance in DENV-infected midguts compared to uninfected controls in at least one time point (Fig 2A and 2B). The Venn diagram shows the numbers of molecular features with differential abundance in the midgut (|log_2_ fold change| ≥ 1 and adjusted p-value < 0.05) upon DENV infection on days 3, 7, and 11 pbm. The profiles of 5,167 features were unaltered at any day post-infection (Fig 2A and 2B). Among the features with altered levels, 274, 283 and 114 features showed differential abundance only on day 3, 7 or 11 pbm, respectively, whereas 98 features showed differential abundance in all 3 days (Fig 2B).

*Identified metabolites*: About 39% of features (363 out of 936) with differential abundance were identifiable through use of three metabolite databases and the putative identifications were categorized into different metabolite classes. The metabolic relationships between the lipid classes are mapped in Fig 2C. Fatty acyl-CoA can be synthesized *de novo*. It is a precursor that can be modified and incorporated into more complex lipid molecules such as glycerophospholipids, sphingolipids, glycerolipids and sterols. On the other hand, fatty acyl-CoA can be degraded through β-oxidation when cells require energy. The overall trend of lipid molecular levels that changed upon DENV infection is summarized in Fig 2D. A majority of the metabolites were unchanged (had intensity changes that are less than 2-fold upon infection or p ≥ 0.05), while a majority of those that showed greater than 2-fold changes in intensity had higher abundances upon DENV infection (Fig 2D and S1 Table, identified tab). Only 61 out of 363 features (~17%) decreased in abundance during DENV infection.

*Unidentified metabolites*: Interestingly, a large number (~61%) of metabolites that were differentially altered in abundance during DENV infection were unidentified (S1 Table, unidentified tab). A majority of these unidentified metabolites had elevated abundance in the DENV-infected samples compared to uninfected controls at all three time points post-infection. These metabolites represent a potential resource since they highlight molecules that are not found in currently available databases that include animal, plant, bacterial and fungal metabolites as well as synthetic compounds. Therefore, they could be compounds unique to Diptera, mosquitoes or *Aedes* species that are yet to be annotated. Given their importance to DENV infection, they should be exploited in the future as possible transmission-blocking control points.

### Glycerophospholipids (GPs)

**Fig 3:**
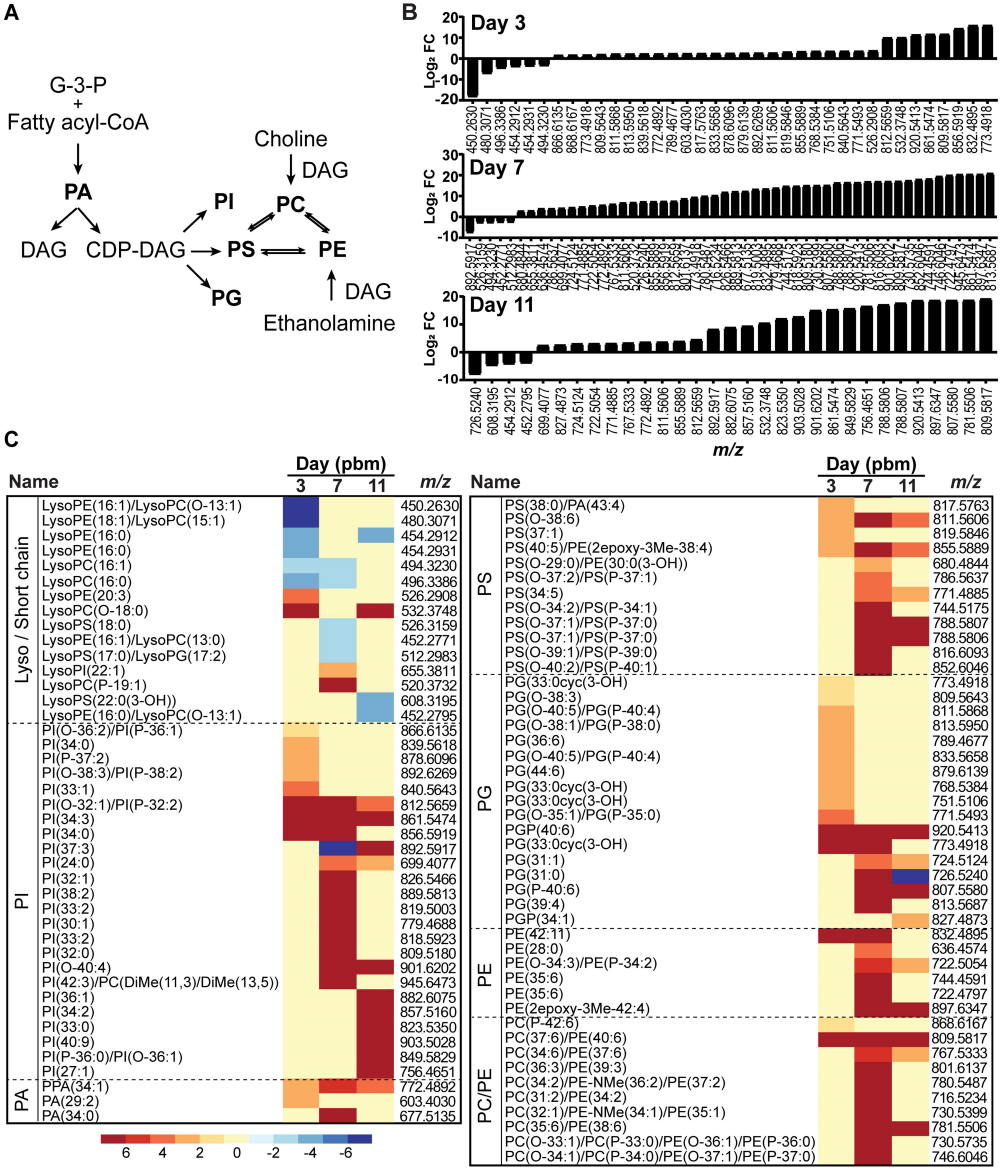
GP fluctuation following DENV infection of mosquito midguts. (A) The metabolic pathway linking GP classes. Highlighted in bold are the GPs observed in this study. (B) GP landscape altered upon DENV infection in midguts. GP species that were altered on days 3, 7, and 11 pbm are listed by *m/z* and arranged by Log_2_ fold changes from lowest to highest. A significant burst of GP abundance was observed at day 7 post-infection coinciding with increased viral replication in the midgut. (C) Heatmap of Log_2_ fold changes of GP species on days 3, 7 and 11 arranged by putative ID subclasses. Abbreviations: CDP-DAG, cytidine diphosphate diacylglycerol; DAG, diacylglycerol; G-3-P, glyceraldehyde-3-phosphate; PA, phosphatidic acid; PC, phosphatidylcholine; PE, phosphatidylethanolamine; PG, phosphatidylglycerol; PI, phosphatidylinositol; PS, phosphatidylserine.

GPs are major components of cellular membranes. GPs are composed of a polar head group, a phosphate group and two fatty acyl chains. The specific composition of GPs in a membrane influences membrane fluidity, leakiness, nutrient exchange, assembly and function of signaling protein complexes and vesicular traffic [18–21]. In insects, the GP composition of membranes also affects tolerance to changing environmental conditions [22]. Pathogenic or endosymbiont infections also drive changes in the intracellular environment and could influence the regulation of GP content in membranes [5, 23–26]. As a point of reference, the metabolic pathways of the major classes of GPs are shown in Fig 3A. Our study identified 565 species of GPs in *Ae*. *aegypti* midguts with 87 species that were altered upon DENV-infection. The alteration landscape and distribution of the different GPs are shown in Fig 3B and 3C.

Minimal enrichment of GP species and levels can be observed on day 3 pbm, during the initial establishment of viral infection in the midgut (Fig 3B). This enrichment was significantly enhanced on days 7 and 11 pbm. These observations were coincident with known viral infection dynamics (peak viral replication in the midgut on day 7 and viral dissemination to other tissues and clearance from the midgut by day 11) [1]. The signaling GPs, PIs, were the most diverse (Fig 3C) closely followed by PGs, important as surfactants and precursors of cardiolipins in mitochondrial membranes. PCs, PEs and PSs, which are primary components of most cellular membranes were also increased during infection especially at the peak of viral replication (day 7 pbm). Interestingly, levels of most of the lyso- or short chain GPs were decreased during infection.

### Glycerolipids (GLs)

**Fig 4:**
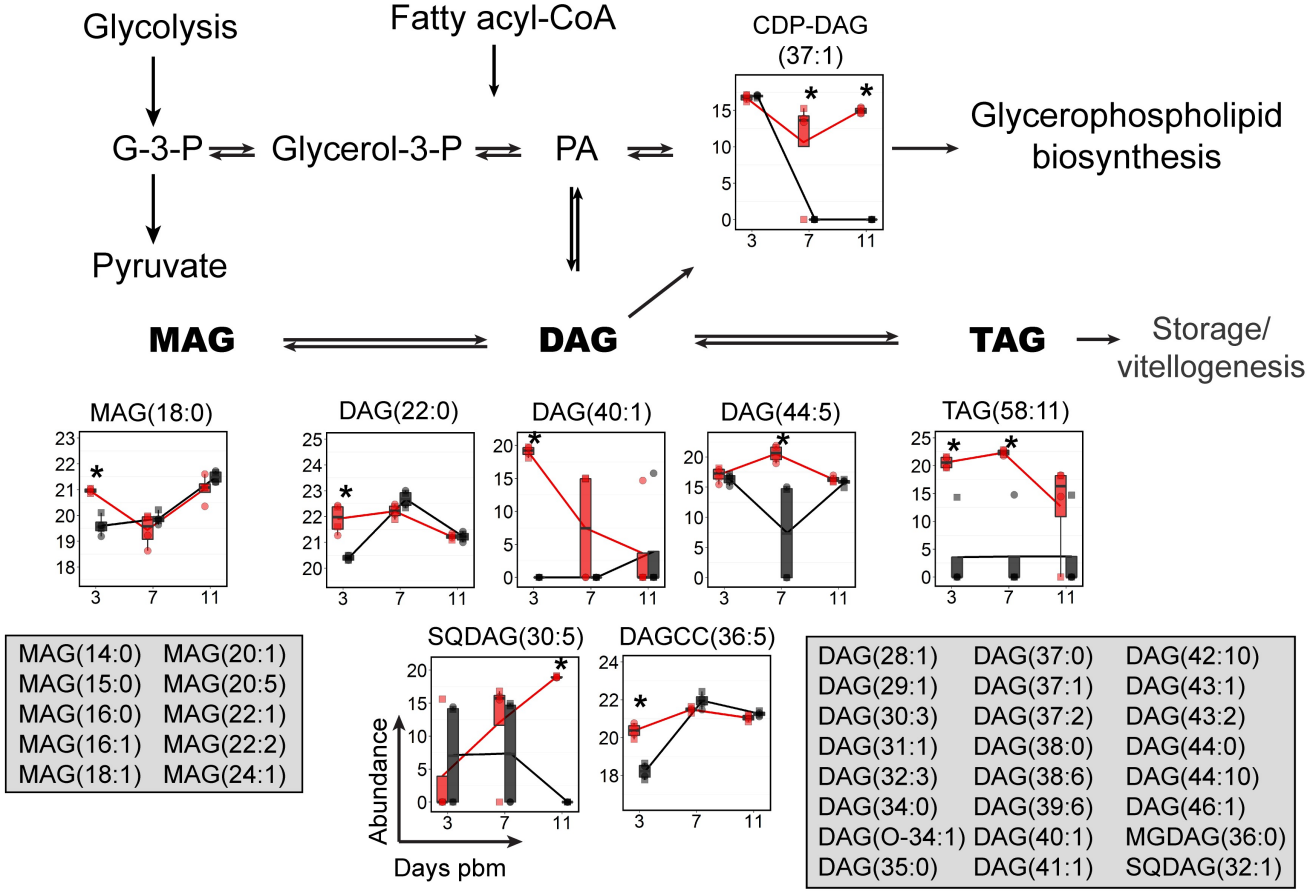
GL levels were dynamically altered upon DENV infection of mosquito midguts. The abundances of GL species detected in mosquito midguts were mapped to the GL biosynthesis pathway. GL molecules that were significantly altered in abundance are shown in boxplots. Feature that were detected but did not change in abundance are listed in the grey boxes. The abundance of metabolites detected in DENV-infected samples is shown in red and uninfected samples in black. Individual sample pools are represented by circles and squares and technical replicates are dots with the same symbol. Asterisk (*) indicates significantly different levels of abundance between DENV-infected and uninfected samples (|log_2_ fold change| ≥ 1, p < 0.05) Abbreviations: DAG, diacylglycerol; CDP-DAG, cytidine diphosphate diacylglycerol; G-3-P, glyceraldehyde-3-phosphate; glycerol-3-P, glycerol-3-phosphate; MAG, monoacylglycerol; PA, phosphatidic acid; TAG, triacylglycerol; SQDAG, Sulfoquinovosyldiacylglycerol and DAGCC, diacylglycerylcarboxy-N-hydroxymethyl-choline.

GLs, including mono-, di- and triacylglycerols (MAG, DAG and TAG), are critical effectors of energy metabolism in insects and mammals. Fatty acids absorbed from a digested blood meal or synthesized *de novo* from a sugar meal can be converted into DAG and TAG that can be transported to the fat body for energy storage or the ovaries for vitellogenesis [27–31] (Fig 4). DAG is also an important intermediate in GP synthesis [18, 32] and a critical second messenger regulating cell proliferation, survival, mitochondrial physiology, gene expression and apoptosis [33, 34]. Seventy species of GLs were identified in our study, of which eight GLs were significantly changed in abundance upon infection. Most of the MAG, DAG and TAG levels were higher during early time points (day 3 and 7 pbm) in DENV-infected midguts (Fig 4). This might be a result of transportation of these lipids from storage tissues to support the demand for lipids during infection [35, 36]. Interestingly, the level of cytidinediphosphate (CDP-DAG (37:1)) were elevated only during the later phases of infection (days 7 and 11 pbm). CDP-DAG is a precursor for GP synthesis (Fig 3A). As a result, increased CDP-DAG (37:1) might support the high demand for GP synthesis on days 7 and 11 pbm during virus infection.

### Sphingolipids (SPs)

**Fig 5:**
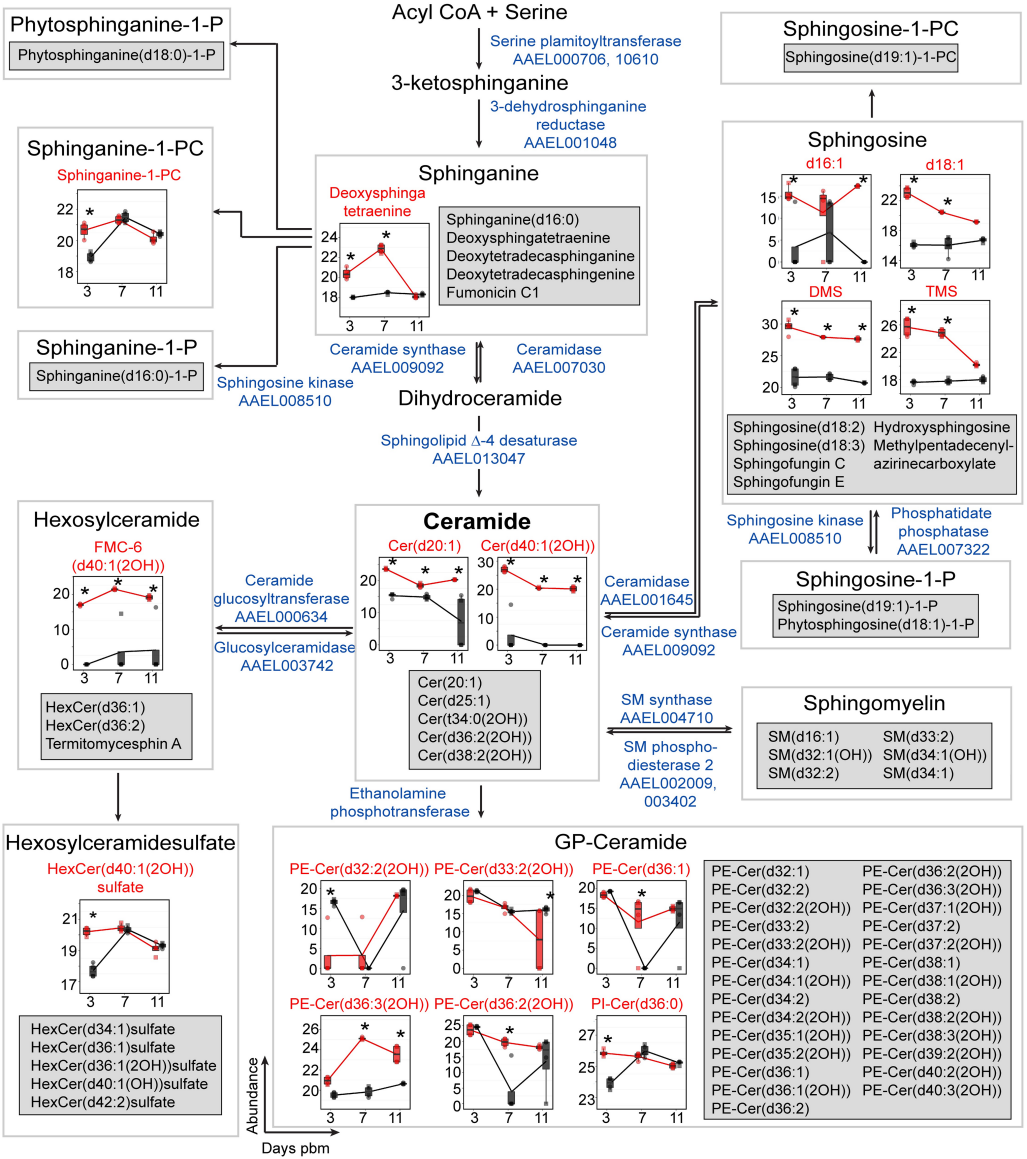
Accumulation of SPs during DENV infection. The SP pathway for *Ae*. *aegypti* was reconstructed for the first time in this study. This SP metabolic pathway (modified from Hannun et al., 2008 [38]) is shown overlaid with box plots with SPs that exhibited significantly altered levels during infection. Features that were detected but unchanged in abundance upon DENV infection are listed in the grey boxes. The abundance of metabolites detected in DENV-infected samples is shown in red and uninfected samples in black. Individual sample pools are represented by circles and squares and technical replicates are dots with the same symbol. Asterisks (*) indicates significantly different levels of abundance between DENV-infected and uninfected samples (|log_2_ fold change| ≥ 1, p < 0.05). The enzymes predicted to catalyze the reactions in the SP pathway and their VectorBase accession numbers (AAELXXXXXX) according to AaegEL5 assembly and as annotated in the KEGG pathway are given in blue text. Where enzymes are not given it indicates an absence of annotation for *Ae*. *aegypti* of names via the KEGG database and VectorBase. Abbreviations: Cer, ceramide; GP-Cer, glycerophospholipid-ceramide; HexCer, hexosylceramide; FMC-6, acetyl-sphingosine-tetraacetyl-GalCer(d40:1(2OH)); PE-Cer, phosphatidylethanolamine-ceramide; Phytosphinganine-1-P, phytosphinganine-1-phosphate; PI-Cer, phosphatidylinositol-ceramide; Sphingosine-1-P, sphingosine-1-phosphate; Sphingosince-1-PC, sphingosine-1-phosphatidylcholine and SM, sphingomyelin.

SPs are bioactive molecules that play important roles in the structural composition of cellular membranes and numerous cell-signaling pathways and are critical in microbial pathogenesis [22, 37–40]. *De novo* synthesis of SPs occurs in the endoplasmic reticulum through the condensation of L-serine and palmitoyl-CoA to form ceramide (Cer) via several intermediates [41, 42]. Cer is a precursor for the biosynthesis of several complex SPs such as sphingomyelin (SM) and glycosylceramides or the production of fatty acids and sphingosines through its hydrolysis [43–45]. The SP pathway has been highlighted in many studies in mammalian cells [37] and in mosquito cells [5] as a lipid metabolic pathway altered by flavivirus infection. In this study, using the identified SPs from both infected and uninfected mosquito tissues we reconstructed a significant proportion of the SP metabolic pathway for *Ae*. *aegypti* (Fig 5). Several members of the pathway were identified at all three time points in both biological replicates of the infected and uninfected tissues. We observed accumulations of several SPs such as sphinganine, sphinganine-1-PC, sphingosine, Cer and hexosylceramide that were elevated in DENV-infected midguts on days 3, 7 and/or 11 pbm. GP-Cers, especially PE-Cers, are sphingomyelin analogues. They function as the principal membrane sphingolipids in *Drosophila*, which lacks sphingomyelin synthase [46]. In mosquito midguts, we found decreased abundance of PE-Cer (d32:2(2OH)) upon DENV infection on day 3 pbm but an increase in the levels of PE-Cer(d33:2(2OH)), PE-Cer(d36:1), PE-Cer(d36:3(2OH)) and PE-Cer(d36:2(2OH)) on day 7 and/or 11 pbm. Interestingly, sphingomyelin was found in *Ae*. *aegypti* mosquito midguts and in cells in culture (Fig 5 and S2–S4 Figs); however, the levels of sphingomyelin were not changed upon DENV infection at any of the time points tested. Glycosylated ceramides (hexosylceramide and hexosylceramidesulfate) play critical roles in modulating cellular signaling and gene expression resulting in changes in processes such as proliferation, apoptosis, autophagy and endocytosis [47]. The abundance of the neutral glycosphingolipid, FMC-6(d40:1(2-OH)), was increased on all days and HexCer(d40:1(2OH) sulfate was increased on day 3 following DENV infection (Fig 5).

### Changes in the Cer-DHCer balance impair DENV infection

**Fig 6:**
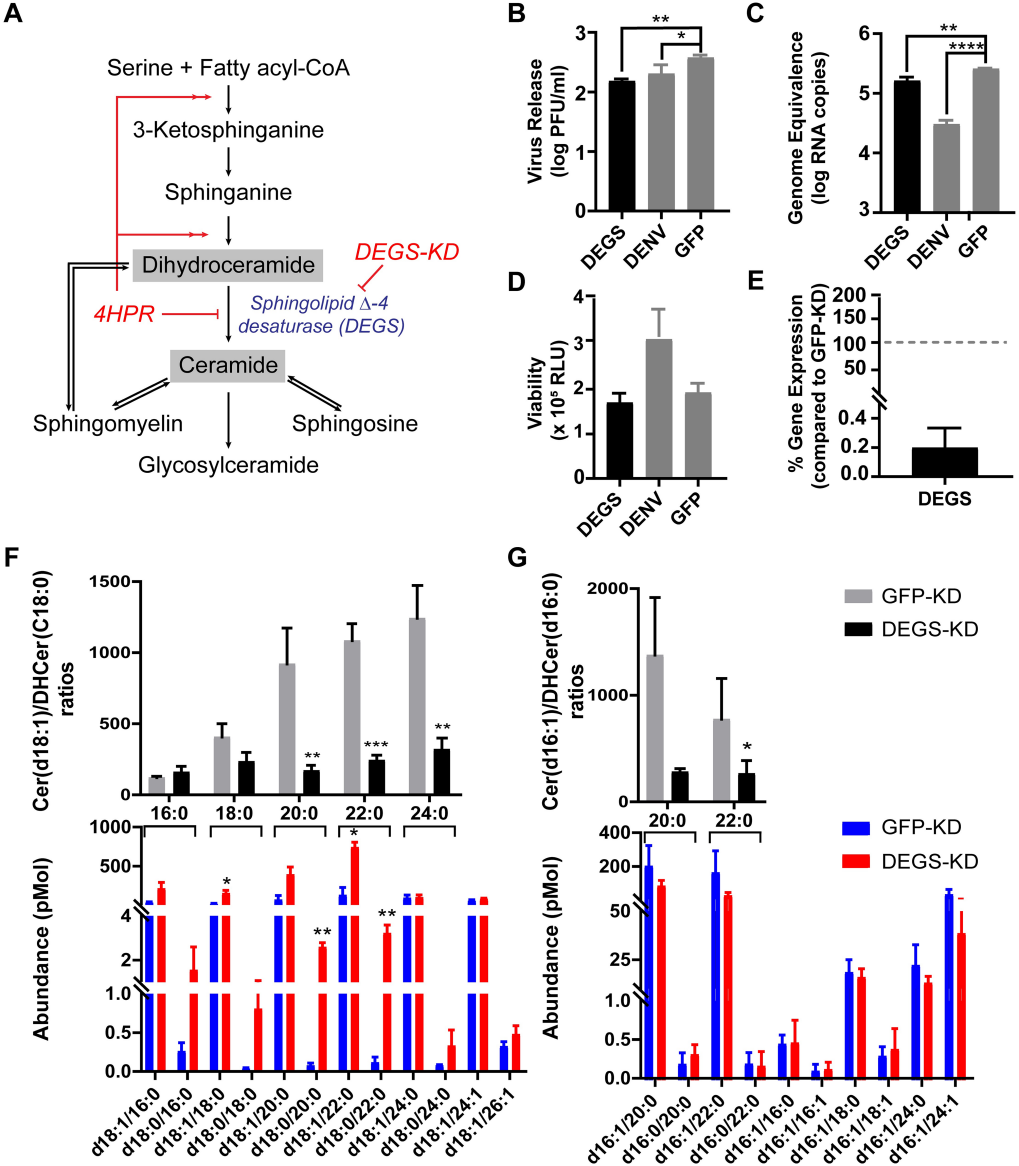
Changes in the Cer-DHCer balance impair DENV infection in Aag2 cells. (A) A schematic of the SP biosynthesis pathway showing the precursor (DHCer) and product (Cer) of the sphingolipid Δ-4 desaturase (DEGS) enzyme-catalyzed reaction. In this experiment, the Cer/DHCer balance was changed by dsRNA knockdown of the *DEGS* gene expression as well as pharmacological inhibition of DEGS with 4HPR (S2 Fig). (B and C) Aag2 cells were transfected with dsRNA derived from *DEGS*, DENV (positive control) or GFP (negative control) genes. Two days post transfection, cells were infected with DENV at 0.3 MOI. Cell culture supernatant (B) or intracellular total RNA (C) were collected at 24 hours post infection (hpi) and analyzed for infectious virus in medium and intracellular DENV RNA, respectively. One-way ANOVA followed by Dunnett’s multiple comparisons test were used for statistical analysis. (D) Viability of Aag2 cells after dsRNA treatments at 48 h post dsRNA transfection. (E) DEGS mRNA expression in DEGS-KD cells compared to the expression of the *DEGS* mRNA in GFP-KD cells (set at a 100%). (F and G) Analysis of Cer and DHCer species in DEGS-KD (red) and GFP-KD cells (blue) using LC-MS/MS Multiple Reaction Monitoring (N = 3). (F) and (G) shows Cer and DHCer molecules with 18- and 16-carbon long chain sphingoid bases, respectively. Lower panels show the abundance of individual species of Cer(d18:1/xx:x or d16:1/xx:x) or DHCer(d18:0/xx:x or d16:0/xx:x) where xx:x refers to the fatty acyl chain attached to each sphingoid head group. The upper panels show the ratio of Cer/DHCer species with the same fatty acyl chain lengths. These ratios highlight the Cer-DHCer imbalance caused by manipulation of DEGS gene expression. Student’s t-test was applied for statistical analysis. * *p* < 0.05, ** *p* < 0.01, *** *p* < 0.005, **** *p* < 0.001.

In this study, we identified a large number of SPs with significantly altered abundance following DENV infection of mosquito midguts. Important biological roles of the SP pathway are determined by conversion from DHCer to Cer, the central precursor of diverse SPs. The balance between Cer and DHCer concentrations plays a critical role in physiologically regulating properties such as membrane architecture, fluidity and function [48]. Therefore, we investigated if alteration of Cer or DHCer levels and the Cer/DHCer ratio had an effect on DENV infection, using *Ae*. *aegypti*-derived (Aag2*)* cultured cells, which are readily amenable to loss of function studies. Cells were treated with 4-hydroxyphenyl retinamide (4HPR) or long double stranded RNA (dsRNA) to inhibit activity of or knock down expression of sphingolipid Δ-4 desaturase (DEGS, VectorBase: AAEL013047, NCBI:LOC5577178), the enzyme that catalyzes conversion of DHCer to Cer, the final step in the *de novo* biosynthesis of Cer (Fig 5), and thus should greatly influence the Cer/DHCer ratio [49]. 4HPR is a synthetic retinoid that activates the Cer *de novo* synthesis pathway prior to DHCer synthesis, but inhibits the activity of DEGS [50] (Fig 6A). Significant reduction of virus titer and genome replication were observed upon 4HPR treatment of Aag2 cells at non-cytotoxic concentrations (S2A–S2C Fig). We validated the metabolic impact of 4HPR on sphingolipid metabolism using multiple reaction monitoring (MRM) LC-MS/MS. We observed accumulation of Cer(d18:1/16:0), Cer(d18:1/18:0), Cer(d18:1/24:1), and Cer(d18:1/26:1) and DHCer(d18:0/18:0) in cells following 4HPR treatment (S2D Fig, lower). However, their accumulation did not alter the Cer/DHCer ratios (S2D Fig, upper). None of the levels of long chain sphingoid bases Cer and DHCer with 16-carbon backbones were affected (S2E Fig). We also did not see changes in sphingosine (d18:1), sphingosine-1-P (d18:1-P), sphinganine (d18:0) or SM levels following 4HPR treatment which is expected as the enzymes that produces these molecules are not the primary targets of 4HPR (S2F and S2G Fig). The effect of 4HPR on DENV replication therefore might not be due to a direct effect on the Cer/DHCer ratios, but due to off target effects such as ROS, changes in bcl-2, p53 mRNA expression and apoptosis [51]. A similar observation has been made in mammalian cells [50].

To validate that the reduction of DENV replication was caused by the loss of SP metabolites or Cer/DHCer imbalance and not due to off-target effects of the inhibitor, the expression of DEGS in Aag2 cells was transiently knocked down by RNA interference, using long dsRNA (Fig 6B–6E). In *DEGS* knockdown (DEGS-KD) cells, DENV titer and genome replication were significantly reduced compared to the GFP dsRNA (GFP-KD) negative control, but were similar to the DENV dsRNA (DENV-KD) positive control (Fig 6B and 6C). SP metabolic profiling of the DEGS-KD cells compared to GFP-KD cells revealed striking reduction of Cer(d18:1/18:0), DHCer(d18:0/20:0) and DHCer(d18:0/22:0) after DEGS-KD (Fig 6F and 6G, lower panel). Interestingly, DEGS-KD also greatly altered the Cer/DHCer ratio for molecules with 18- and 16-carbon long chain sphingoid bases, especially those with very long fatty acyl chain lengths (20:0–24:0) (Fig 6F and 6G, upper panel). These results indicated that perturbation of the Cer-DHCer balance reduced both DENV genome replication and infectious virus formation. As expected we did not see changes in sphingosine (d18:1), sphingosine-1-P (d18:1-P), sphinganine (d18:0) or SM levels during dsRNA treatment (S3 Fig). When DENV-infected and uninfected Aag2 cells were compared for alterations in the SP pathway, we observed a significant increase in the abundance of Cer(d18:1/20:0) and Cer(d18:1/24:1) and DHCer(d18:0/22:0) in infected cells, but a decrease in Cer(d16:1/16:1) in infected cells (S4A and S4B Fig, lower). These changes did not, however, affect the Cer/DHCer ratio for very long fatty acyl chain containing molecules (S4A and S4B Fig, upper). We did not see changes in sphingosine (d18:1), sphingosine-1-P (d18:1-P), sphinganine (d18:0) or SM levels during dsRNA treatment (S4C and S4D Fig).

### Fatty acids and derivatives (Fatty acyls)

**Fig 7:**
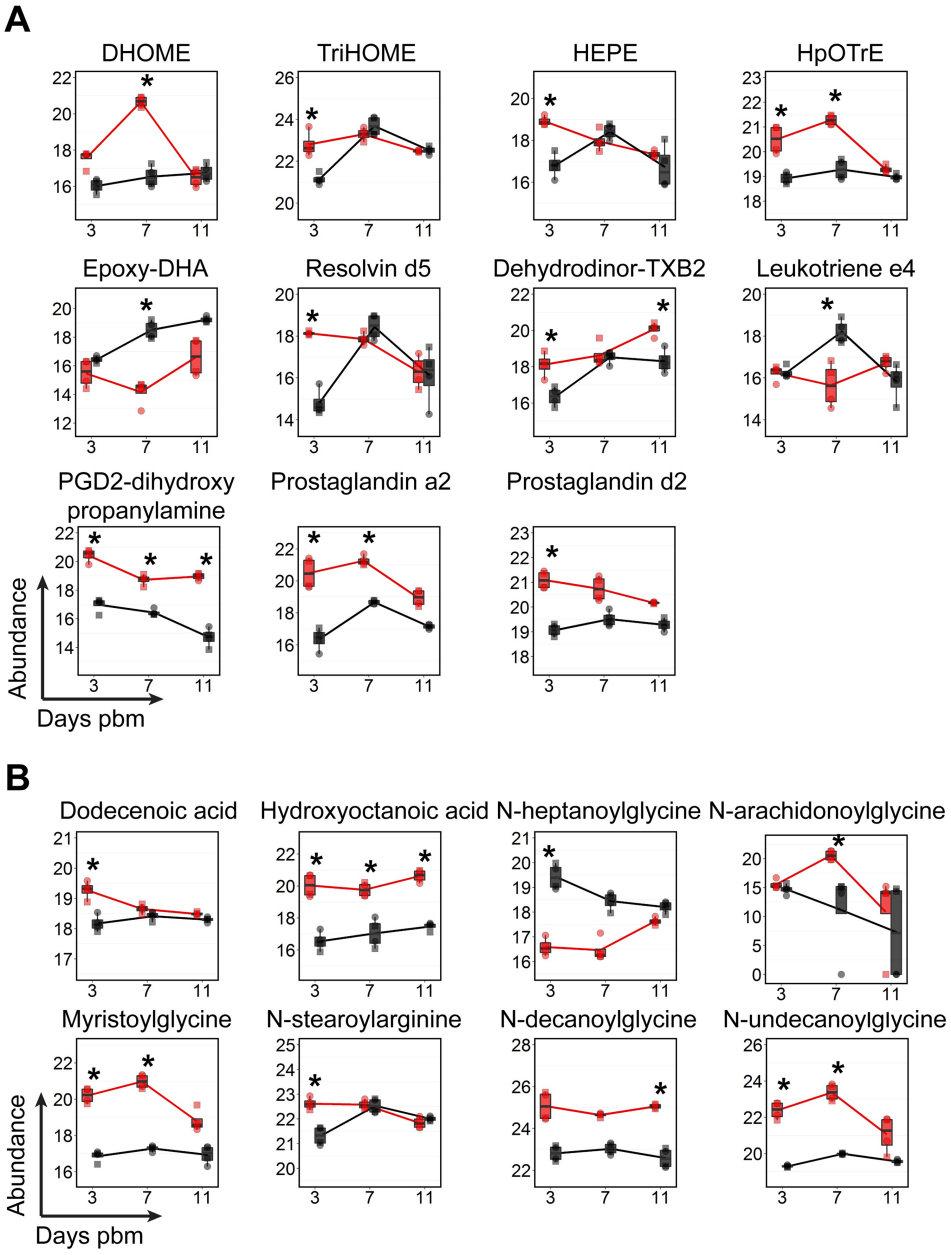
Temporal fluctuations in levels of fatty acyl molecules following DENV infection of mosquito midguts. (A) The abundances of fatty acyls that have known functions in immunomodulation. (B) The abundances of free fatty acids and N-acylamides that have known functions in signaling and/or that are markers of malfunction in fatty acid oxidation are shown. The abundances of metabolites detected in DENV-infected samples is shown in red and uninfected samples in black. Individual sample pools are represented by circles and squares and technical replicates are dots with the same symbol. Asterisks (*) indicates a significantly different abundance between DENV-infected and uninfected samples (|log_2_ fold change| ≥ 1, p < 0.05). Abbreviations: DHOME, dihydroxyoctadecenoic acid; Dehydrodinor-TXB2, dehydrodinor-oxothromboxadienoic acid; Epoxy-DHA, epoxydocosahexaenoic acid; HEPE, hydroxyeicosapentaenoate; HpOTrE, hydroperoxyoctadecatrienoic acid; PGD2-dihydroxypropanylamine, Prostaglandin D2-dihydroxypropanylamine and TriHOME, trihydroxyoctadecenoic acid.

Fatty acyls can be acquired from the midgut digestion and absorption of a blood meal or can be *de novo* synthesized from central carbon metabolism precursors [27, 52]. Fatty acids that are covalently linked to coenzyme A, fatty acyl-CoAs, can be incorporated into complex lipids such as GPs, SPs and GLs that have structural roles in membranes. In addition, free fatty acids or fatty acyls that are linked to other functional groups can have critical roles in signaling, energy homeostasis and the immune response in insects and mammals (e.g.: fatty amides and eicosanoids) [53–55]. In this study, at least nine subclasses of fatty acyls were detected and many of these had higher abundances in DENV-infected midguts compared to uninfected controls in at least one time point pbm (S5 Fig and S1 Table). The molecules observed were fatty amides, hydroxy fatty acids, free fatty acids, eicosanoids and leukotriene (i.e. immunomodulators), fatty-amines, glycosides, dicarboxylic acids, and keto-fatty acids. Fig 7 shows molecules with significantly increased or decreased levels at least at one time point following DENV infection and have known functions in immunomodulation (Fig 7A) and N-acylamides and other fatty acyls whose accumulation is a marker for malfunction of fatty acid oxidation (Fig 7B). Prostaglandins, leukotrienes, thromboxanes and lipoxins are eicosanoids that are synthesized in various tissues. Eicosanoids are inflammatory mediator molecules have been found to play several important roles in insect immunity [55]. In this study, three prostaglandins (prostaglandin a2, prostaglandin d2, and PGD2-dihydroxypropanylamine) and one thromboxane (dehydrodinor-TXB2) showed increased levels during DENV infection and one leukotriene (leukotriene e4) decreased during DENV infection. Anti-inflammatory molecules (resolvin d5 and epoxy-DHA) increased during DENV infection [56, 57]. Metabolites of linoleic acid that may regulate prostaglandin synthesis (HEPE, HpOTrE and TriHOME) also increased upon DENV infection [58]. N-acylamides play important roles in endocannabinoid signaling systems in mammals and have been detected and reported as potentially important signaling molecules in *Drosophila* [59]. Elevated levels of these molecules are also observed in the urine and blood of human patients with fatty acid oxidation disorders [60]. In our study, we found that five N-acylamide molecules (e.g. N-arachidonoylglycine, myristoylglycine, N-stearoylarginine, N-decanoylglycine, and N-undecanoylglycine) had increased levels and N-heptanoylglycine displayed a decreased level in midguts during DENV infection (Fig 7B).

### Acyl-carnitines

**Fig 8:**
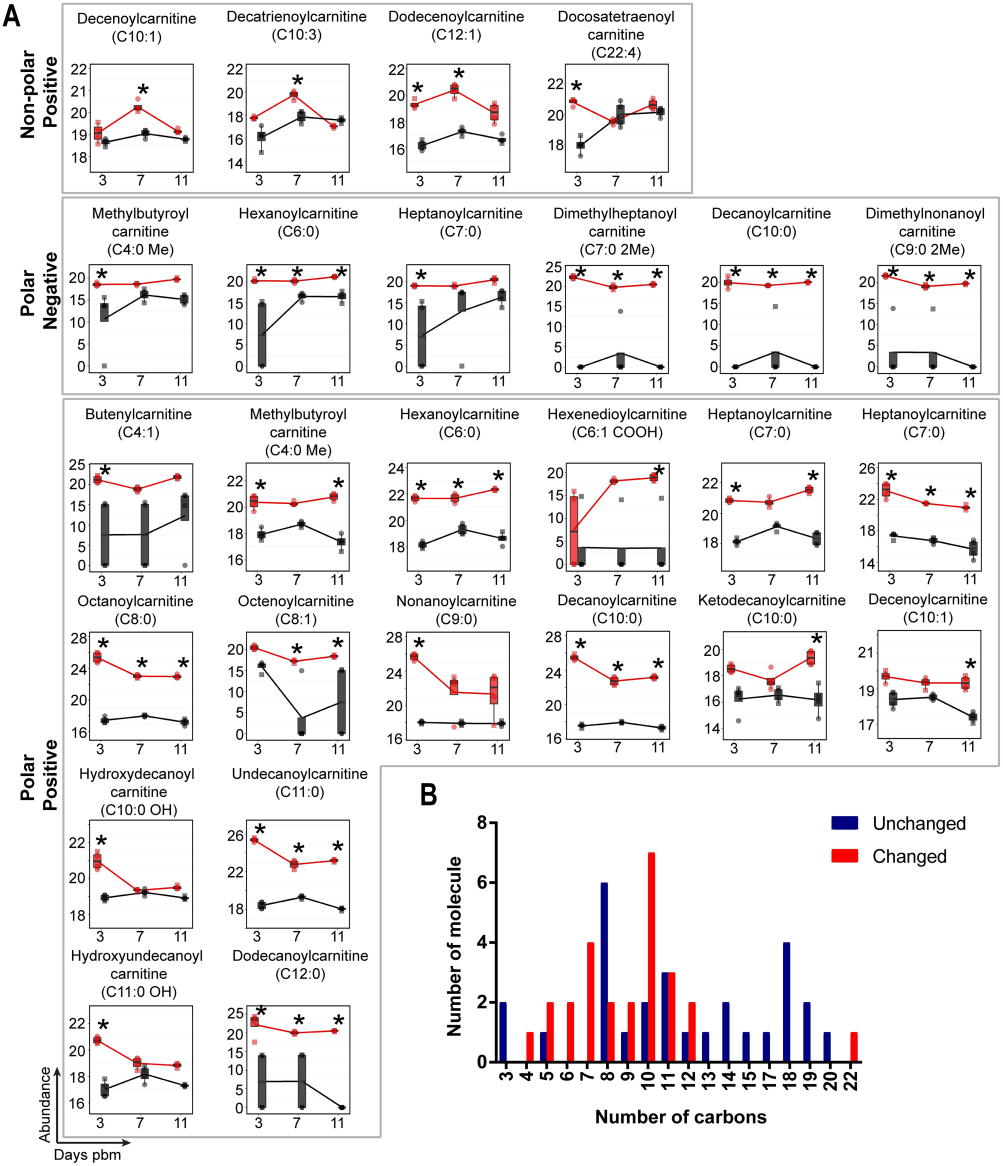
Acyl-carnitines accumulate in mosquito midguts during DENV infection. (A) Molecules showing differential abundance after DENV infection. Numbers of carbons in the fatty acyl chains are indicated in parentheses. The abundance of metabolites detected in DENV-infected samples is shown in red and uninfected samples in black. Individual sample pools are represented by circles and squares and technical replicates are dots with the same symbol. Asterisks (*) indicates significantly different abundance between DENV-infected and uninfected samples (|log_2_ fold change| ≥ 1, p < 0.05). (B) Bar graph showing numbers of detected acyl-carnitine molecules corresponding to the numbers of carbons in the acyl-chains. Red bars indicate molecules with significantly altered abundances while blue bars indicate molecules that remained unaltered during DENV infection.

One of the most prominent observations in this study is the change in abundance of acyl-carnitines in midgut tissue after DENV infection (Fig 8). These molecules are intermediates that shuttle fatty acyl-CoA from the cytoplasm into the mitochondria for β-oxidation and subsequent energy (ATP) production (Fig 8). A total of 54 acyl-carnitine molecules were detected in the midgut metabolome. Following DENV infection, 26 acyl-carnitines had a significant increase in abundance and only one had decreased abundance (Fig 8A). Interestingly, of those that increased in abundance, 25 out of 26 molecules contained medium chain-length fatty acyls of 4–12 carbons. The accumulation of acyl-carnitines with medium-length carbon chains was reported to be a result of incomplete β-oxidation [61].

### Sterol lipids

Sterol lipids are a group of cyclic organic compounds composed of 17 carbon atoms arranged in a four-ring structure. In insects, cholesterol is a component of cellular membranes and a precursor of the ecdysone hormone [15, 62–65]. However, insects cannot synthesize cholesterol *de novo* but must absorb it from dietary sources and/or microflora [15, 16, 66]. During infection of mammalian cells, cholesterol in the flavivirus envelope seems to be critical for viral entry and fusion [67, 68]. It has also been shown that flaviviruses can manipulate cellular cholesterol homeostasis to facilitate genome replication [69]. However, the role of cholesterol in flavivirus replication in the mosquito vector has not been determined. In this study, we detected 111 sterol lipid molecules. Many of these have no reported function in insects. Since sterols in the mosquito are acquired from dietary sources (in this case, raisins, sucrose, sheep’s blood and cell culture medium), we did not exclude sterol lipids identified as plant or other animal from our analyses. Of the 111 molecules, 25 showed different levels of abundance during DENV infection compared to controls (S1 Table). Twenty-one molecules increased during infection while only four molecules decreased during infection. A majority of the changes occurred on day 3 (10 molecules) and day 7 (14 molecules) pbm. Only one molecule showed significant changes (decreased) on day 14 pbm.

## Discussion

**Fig 9:**
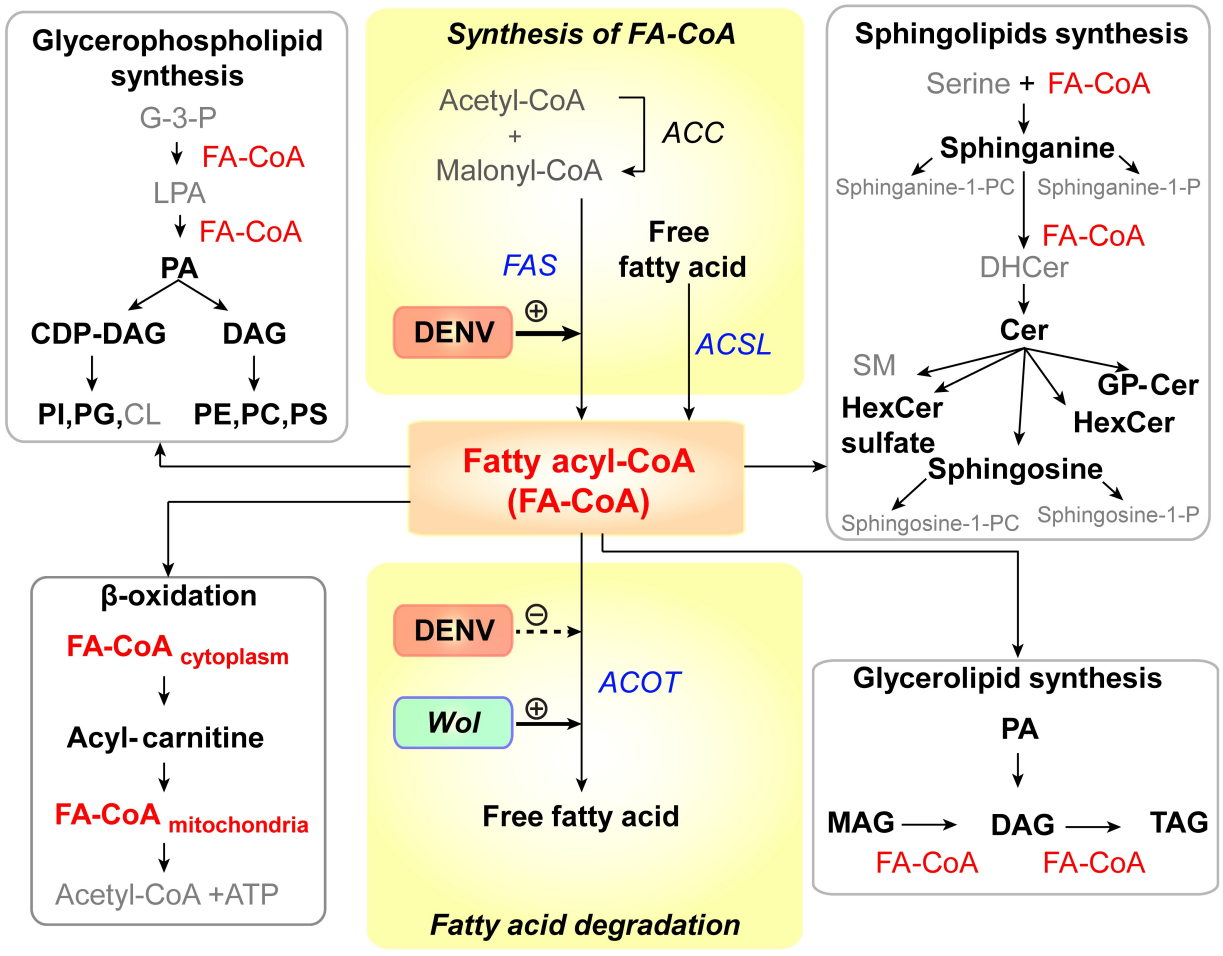
DENV infection results in alteration of lipid homeostasis in infected *Ae*. *aegypti*. This study revealed that the abundance of lipids in six main lipid metabolic pathways [72] was altered following DENV infection of the mosquito midgut (names of lipids that were altered in abundance during infection are in black (bold) and lipids that remained unchanged are in grey). All of these pathways require fatty acyl-CoA (FA-CoA), activated fatty acids, as a precursor for the synthesis of complex lipids (red). Names of enzymes involved in FA-CoA homeostasis are italicized (*FAS*, *ACOT* and *ACSL*). Previous studies by Heaton et al, 2010 [6] and Perera et al, 2012 [5] have shown that DENV infection requires and can enhance the activity of FAS to synthesize in human and mosquito cells. Ye et al, 2013 [73] have shown that *Wolbachia*, the insect endosymbiont used to control DENV transmission by *Ae*. *aegypt*i mosquitoes can increase FA-CoA catabolism by increasing the expression of ACOT enzymes. Therefore, FA-CoA metabolism represents a ‘hub’ that may control lipid metabolic competition in the midgut that mediates the success of DENV replication. Abbreviations: ACC, acetyl-CoA carboxylase; ACSL, long-chain acyl-CoA synthetase; ACOT, acyl-CoA thioesterase; CDP-DAG, cytidine diphosphate diacylglycerol; Cer, ceramide;; CL, cardiolipin; DAG, diacylglycerol; DHCer, dihydroceramide; G-3-P, glycerol -3-phosphate; GP-Cer, glycerophospholipid-ceramide; FAS, fatty acid synthase; HexCer, hexosyl ceramide; LPA, lysophosphatidic acid; MAG, monoacylglycerol; PA, phosphatidic acid; PC, phosphatidylcholine; PE, phosphatidylethanolamine; PI, phosphatidylinositol; PG, phosphatidylglycerol; PS, phosphatidylserine; SM, sphingomyelin; sphinganine-1-P, sphinganine-1-phosphate; sphinganine-1-PC, sphinganine-1-phosphatidylcholine; sphingosine-1-P, sphingosine-1-phosphate; sphingosine-1-PC, sphingosine-1-phosphatidylcholine; TAG, triacylglycerol and *Wol*, *Wolbachia*.

The dynamic metabolic environment of an organism is an indicator of physiological changes that occur following exposure to varying environmental conditions. When arboviruses such as DENV infect a mosquito vector, they must strike a delicate balance between metabolic commensalism and competition to achieve persistent replication in mosquito tissues to allow transmission to a new host. Here, we have explored the metabolic landscape of *Ae*. *aegypti*, the primary vector of DENV, ZIKV, YFV and CHIKV, during infection with DENV. Our study focused on the midgut, the site of initial viral replication prior to viral dissemination to other tissues and transmission to the vertebrate host [1]. The observed biochemical changes highlight specific metabolites that may be markers of infection. They may be required for viral replication or produced as part of the defensive response of the vector to the invading pathogen. We have consistently observed a link between DENV infection and an increased abundance of GPs; our previous work on DENV-infected human cells showed that fatty acid synthase (FAS), a key enzyme required for fatty acid synthesis (essential pre-cursors of GP synthesis), is activated by the expression of virus-encoded nonstructural protein NS3, and relocates to sites of viral RNA replication through interaction with NS3 [6]. Inhibition of FAS activity using C75 caused a reduction in viral replication and we observed this in both cultured human and mosquito cells [5, 6]. We profiled the metabolic changes upon C75 inhibition of FAS in mosquito cells and demonstrated that the GP repertoire was directly reduced coincident with the reduction in viral replication. Therefore, in the cell culture models an abundance of GPs is required for viral replication. The current study supports these observations and reveals that DENV infection also significantly alters the abundance of GPs and other lipid-related molecules in the midgut of the mosquito vector during infection (summarized in Fig 9). While our previous studies showed that *de novo* synthesis of lipids plays an important role in DENV replication, other processes such as lipolysis, lipid conversion/consumption or import/export may also critically shape the metabolite pool observed in these infected tissues [70, 71].

### Glycerophospholipids (GPs)

One of our most striking observation was the marked elevation of GP levels coincident with the kinetics of viral replication in the midgut [1]. Digestion of the blood meal, is completed within ~48 hours post-ingestion, and provides a major source of amino acids and fatty acids used for the biosynthesis of macromolecules [74, 75]. These macromolecules are required for vitellogenesis (yolk formation) and energy storage [29, 76–78]. In DENV-infected mosquitoes, the rapid increase in GP content is observed at day 7 pbm when viral replication reaches its peak in the midgut. While it is currently unknown if biosynthesis alone contributes to this burst in GP content the increase is consistent with requirements for DENV replication within human and mosquito cells in culture [5, 6]. Specifically, DENV infection induces a massive expansion of intracellular membranes required for the assembly and function of viral replication complexes and assembly and release of enveloped progeny virions [4, 7]. This membrane expansion is coincident with increased GP biosynthesis through the co-opted functions of FAS, a key enzyme in the pathway. Electron microscopic studies have revealed similar membrane expansions associated with the midgut of WNV-infected *Culex* mosquitoes but equivalent studies have not been performed in *Ae*. *aegypti* infected with DENV [14].

### Sphingolipids (SPs)

Reconstruction of the SP pathway in *Ae*. *aegypti* revealed significant conservation with that of *Homo sapiens*, allowing us to identify a majority of orthologous metabolites in the pathway. Importantly, most metabolites were identified in both pools of 100 midguts, at all time points and in both infected and uninfected tissues confirming a significant presence in the midgut metabolome. Additionally, we were also able to identify orthologous effector enzymes for a majority of the SP metabolic pathways in our study using annotations in the AaegEL5 assembly of the *Ae*. *aegypti* genome on VectorBase [79]. SPs are important components of lipid rafts in membranes that in mammalian systems, together with cholesterol support the assembly and function of various protein signaling complexes [80]. Metabolites of SPs are well-studied signaling molecules [41]. These lipids have been studied in *Drosophila* [23, 81, 82] and it has been demonstrated that these lipids are critical for development, reproduction and maintenance of tissue integrity in the fruit fly [83]. SPs have also been identified in *Anopheles stephensi* by spatial mapping [84] and in *Aedes* [5] and *Culex* cells in culture [85]. However, the specific functionality of these lipids has not been extensively studied either *in vitro* or *in vivo* in mosquitoes. All viruses and most bacteria are incapable of SP synthesis and rely on the host to provide these lipids. The specific species of SPs that are increased in abundance during infection could influence the balance between commensalism, competition and/or host damage [37, 86].

In our data, the most striking observation is that several SP metabolites that are precursors or derivatives of Cer showed increased abundance in DENV-infected midguts compared to controls. In *Ae*. *aegypti-*derived cells we observed a significant increase in the abundance of select Cer and DHCer species and a reduction in other Cer species during infection. Importantly, altering the ratio of Cer to DHCer (through inhibition of DEGS) significantly reduced viral genome replication and infectious virus release, highlighting the importance of this hub for infection. In our previous studies in C6/36 *Ae*. *albopictus* mosquito cells, we also observed elevated levels of Cer and SM during DENV infection [5]. An important observation from the three systems studied thus far (*Ae*. *aegypti* mosquito midgut tissue, *Ae*. *aegypti*-derived Aag2 cells and *Ae*. *albopictus*-derived C6/36 cells) is that the SP synthetic pathway is significantly activated during infection, with the Cer concentration being a focal point. In contrast, work by Aktepe et. al., on mammalian cells showed that DENV replication was enhanced upon the inhibition of Cer synthesis [86]. Therefore, while a critical focal point during infection, the SP synthetic pathway requires further investigation to determine how the Cer hub and other intermediates in the SP metabolic pathway might support or limit infection.

### Mitochondrial β-oxidation and metabolite trapping

During mitochondrial β-oxidation activated fatty acids (FA-CoAs) in the cytoplasm are esterified with carnitine to produce acyl-carnitines that are then shuttled to the mitochondrial matrix for energy production [87–89]. In this study, we observed a temporal accumulation of medium-length carbon chain-containing acyl-carnitines in DENV-infected midguts compared to uninfected midguts. The accumulation of medium-length carbon chain acyl-carnitines was reported to be indicative of incomplete β-oxidation [90]. Given that DENV replication requires FA-CoAs, for lipid biosynthesis and subsequent membrane expansion, it is possible that acyl-carnitine accumulation results from a manipulation of β-oxidation (an increase or decrease) in order to provide for viral replicative needs [5, 71, 86].

**Fig 10:**
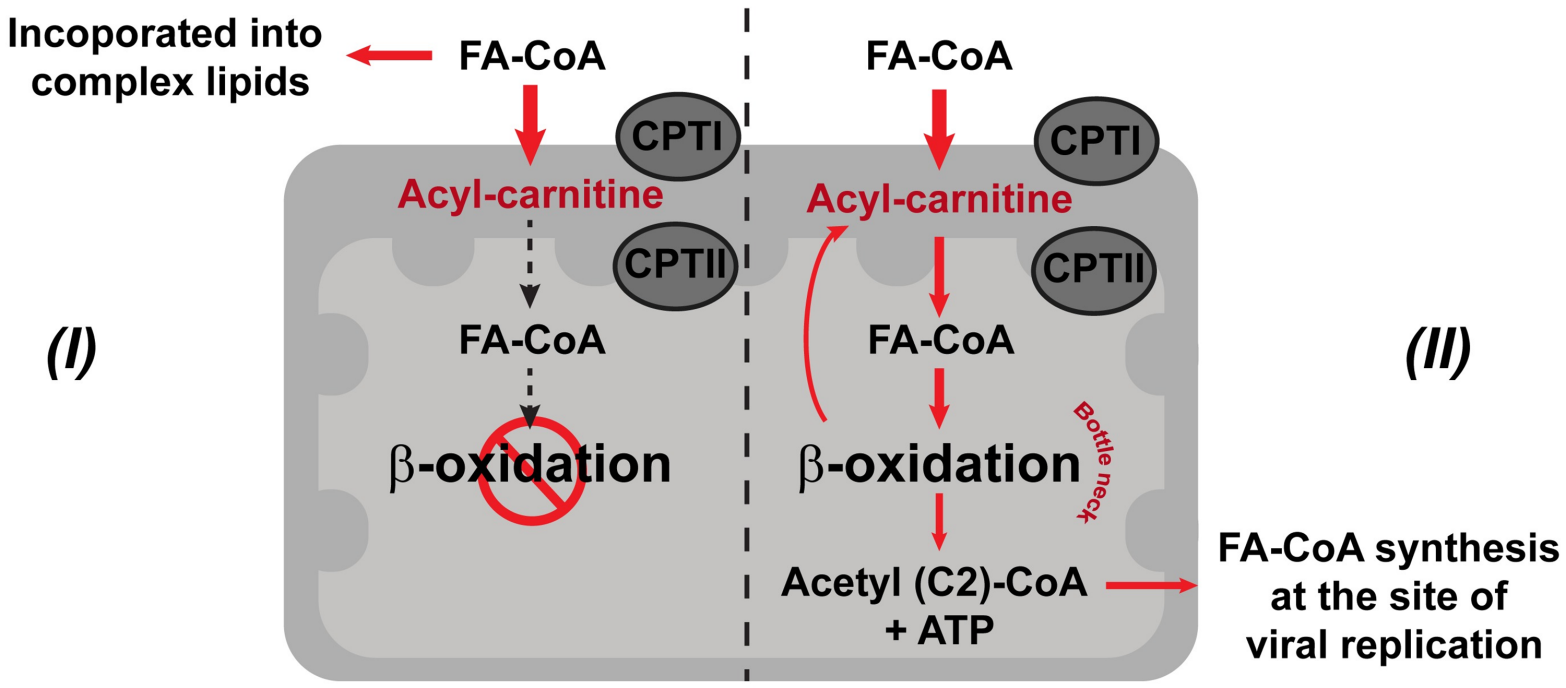
DENV infection perturbs cellular energy production from lipids. The schematic shows the carnitine shuttle translocating fatty acyl-CoA from the cytosol into the mitochondrial matrix for fatty acid degradation (β-oxidation) and two hypotheses (*I and II*) to explain the accumulation of medium-chain length acyl-carnitines and the diversion of FA-CoAs during DENV infection. Hypothesis *I* represents a pathway where an accumulation of acyl-carnitines is observed because β-oxidation in the mitochondria is inhibited or blocked by infection. Hypothesis *II* represents a pathway that leads to mitochondrial overload during infection due to increased energetic demands. Abbreviations: CPTI, carnitine palmitoyl transferase I; CPTII, carnitine palmitoyl transferase II; and FA-CoA, fatty acyl-CoA. Red arrows represent the hypothesized flow of intermediates occurring during infection.

There are two possible hypotheses to explain the accumulation of medium-chain length acyl-carnitines during DENV infection (Fig 10). In hypothesis I, accumulation of acyl-carnitines might be caused by inhibition or blockage of β-oxidation in the mitochondria reducing the production of ATP (Fig 10*I*). This phenomenon has been observed in cells exposed to hypoxic conditions [91–93]. Similar effects were observed during DENV infection of human cells; in infected HepG2 cells, mitochondrial membrane damage appeared to lead to a reduction in ATP levels [94]. To maintain energy homeostasis, energy consumption is diverted towards increasing glucose uptake and glycolysis [91]. This response has also been observed in DENV-infected primary human cells [95]. Due to the stalling of acyl-carnitine transport and a blockage of β-oxidation, FA-CoA lipid partitioning may occur to divert FA-CoAs towards a synthesis of complex lipids and membrane expansion at the expense of fatty acid oxidation [92, 95, 96].

Alternatively, in hypothesis II, an accumulation of medium-length carbon chain acyl-carnitines could occur because of mitochondrial overload (Fig 10*II*). In this scenario, due to increased energetic demands during infection, a large proportion of FA-CoAs enter mitochondria but are only partially broken down by β-oxidation, which serves as a bottle neck for this reaction. This phenomenon was observed in human skeletal muscle insulin resistance [61]. The ATP that is produced by β-oxidation is sufficient to supply the energetic needs for producing more virions, and acetyl-CoA (2 carbon product) can be recycled for *de novo* synthesis of new longer-chain FA-CoA molecules directly at the site of virus replication [6]. This hypothesis is supported by Heaton et al. during DENV infection of Huh7.5 human liver cells, where they observed an increase in autophagosome processing of cellular lipid droplets and TAGs to produce free fatty acids and a stimulation of mitochondrial β-oxidation [71]. The molecular mechanisms of how mitochondrial energetics are diverted to benefit DENV replication in the mosquito midgut are still unclear and the observations in this study present the first insight into the possibilities.

### Metabolic competition versus commensalism

FA-CoAs (activated fatty acids) form a primary hub that integrates multiple lipid metabolic pathways, some of which were found to be perturbed in this study during DENV infection (Fig 9). These molecules are key to the synthesis of more complex lipids, which are important for DENV replication in both human and mosquito cells [4–7]. FA-CoAs are also important for activating several signaling pathways in the cell [97, 98]. Interestingly, there is evidence that *Wolbachia*, the endosymbiotic bacterium used to control DENV infection in *Ae*. *aegypti*, might drive this metabolic balance in the opposite direction (FA-CoA to free fatty acid) suggesting opposing metabolic driving forces resulting in a competition between virus and endosymbiont [73]. Recent studies in *Wolbachia*-infected, *Ae*. *albopictus* (Aa23) cells have supported this hypothesis and indicated that several lipid groups such as SP, PC, and DAG previously shown to be elevated in DENV-infected C6/36 cells were depleted upon *Wolbachia* infection of the cells [5, 23, 24].

The hypothesis that metabolic challenge rather than immune pathways form the basis for pathogen-blocking capabilities of *Wolbachia is* further supported by observations by Caragata et al, 2013 [99] who showed that cholesterol might also be a limiting factor that drives metabolic competition between virus and endosymbiont. Specifically, increasing cholesterol availability via an enriched diet increases virus replication in the insect and reduces the impact of *Wolbachia*-mediated pathogen suppression. Mosquitoes are incapable of *de novo* synthesis of sterols [16, 100], and must sequester these molecules from the blood meal or from microbiota that are capable of synthesizing these lipids. In the presence of *Wolbachia*, it is possible that cholesterol “stealing” from the vertebrate blood meal may make cholesterol unavailable for DENV infection/replication. In mammalian systems, it has been shown that cholesterol is critical for DENV replication [67, 68], and therefore, it is interesting to contemplate what might happen in the mosquito where these lipids are limited. Alternately, other species of lipids could substitute for the function(s) of cholesterol during DENV replication in the mosquitoes. In this study, we detected over hundred cholesterol molecules with one fourth of the molecules showing alterations during early phases of DENV replication in the midgut. The specific role these molecules might play in the virus-vector interactions yet remain to be explored.

### Comparison to cell culture models

These *in vivo* studies are the obvious next step to previous studies that have evaluated metabolic changes induced by DENV infection in cell culture models. Interestingly, in both human [6] and mosquito cell cultures models [5] we observed a significant increase in GP expression during DENV infection. We determined that this burst of GP abundance is due to the activity of FAS that seems to be stimulated and re-localized to sites of viral RNA replication by DENV nonstructural protein 3 (NS3) [5]. We anticipated that this might also be true *in vivo*. Accordingly, we saw a significant increase in GPs in *Ae*. *aegypti* midguts that directly coincides with the peak of viral replication (similar to that observed in both human hepatoma and C6/36 cells) indicating that it could be a key modulator of DENV infection. In mosquitoes, *in vitro* (C6/36 cells) we did not see significant PG/PI involvement the during infection. However, we do see activity in mosquito midguts following infection. The concentrations of PG lipids are reportedly lower in eukaryotic membranes but higher in bacterial membranes, and therefore could be elevated in the midguts by contributions from the microbiome [101]. However, to accurately compare the specific GP landscape with respect to true concentrations of head groups, extensive targeted analyses will need to be carried out in the future. Lysophospholipids (LysoGPs) also seemed more heavily perturbed in C6/36 cells compared to mosquito midguts with evidence that phospholipase A2 may have increased activity during DENV infection [5]. A striking observation in this study is that among the GPs that were significantly altered in abundance during infection, lysoGPs were prominently decreased in abundance in infected midguts while they were increased in abundance in infected C6/36 cells. These lipids are known to be stress-related as well as to function as precursors and signaling molecules that also play important roles in influencing membrane architecture [102, 103]. These different response patterns *in vitro* and *in vivo* indicate a more complex situation in the whole animal and is evidence that *in vitro* observations are not always translatable to the *in vivo environment*.

Sphingolipids (SPs) also showed differences between the cell culture and *in vivo* systems. While in C6/36 cells some of the major SPs like SM and Cer were perturbed during infection, in the midgut tissues, we saw both precursors and downstream products of Cer metabolism as well as complex SPs being perturbed. Interestingly, while several SMs were detected, we did not see any significant changes in SM abundance during infection. The observation of SPs in Aag2 cells was rather uninformative, with only a few Cer and DHCer molecules showing significant differences in abundance during infection. However, alteration of the Cer to DHCer ratio in these cells by RNAi knockdown significantly reduced DENV genome replication and infectious virus release. These observations suggested that there may be a battle between the virus and the host to regulate the Cer hub that results in keeping most of the SPs in balance. Actively changing this hub can significantly impact the viral life cycle. A caveat is that when comparing metabolic responses of these different *in vivo* and *in vitro* models systems to DENV infection, it is important to take into account the inherent differences between the systems that might impact this response. For example, C6/36 cells, are a cell line derived from *Ae*. *albopictus* larvae (a different species to *Ae*. *aegypti*), and are defective in the RNAi pathway [104]. The Aag2 cell line, while derived from *Ae*. *aegypti* embryos are persistently infected with cell fusing agent virus (CFAV) [105]. Our *in vivo* studies were carried out in adult female *Ae*. *aegypti* midgut tissue, representing a much more complex and biologically relevant cellular environment.

### Summary

We present the first comprehensive analysis of the metabolome of *Ae*. *aegypti*, the primary mosquito vector of dengue, Zika, chikungunya and yellow fever viruses, during DENV infection. Alterations in the metabolome are quantitative indicators of the outcome of arbovirus-host interactions that facilitate virus replication or may result from host responses to infection. Our study provides evidence that DENV infection specifically alters the lipid repertoire at the initial site of viral replication, the midgut of the mosquito. Since this study was carried out using a highly susceptible mosquito strain with a laboratory-adapted virus, it highlights metabolic pathways that may be critical for achieving optimal levels of replication in the midgut required for successful dissemination and ultimately transmission. Given that vector competence for infection and transmission is dependent on the specific virus-vector pairing, it will be critical to evaluate the metabolic environment under conditions where the virus has a less robust infection rate and/or the vector presents infection and transmission barriers. This is particularly important in field settings where vector competence is also influenced by environmental conditions, co-infecting pathogens and the microbiome of the vector. In addition, there is a growing body of evidence that metabolic competition between arboviruses and endosymbionts such as *Wolbachia* may form the basis of endosymbiont-mediated control of viral transmission. Therefore, exploring vector metabolism is a powerful and integrative means to identify metabolic control points that could be exploited to interfere with pathogen transmission or to implement vector control.

## Materials and methods

### Virus

DENV serotype-2 strain Jamaica 1409 (JAM-1409) was obtained from the Centers for Disease Controls and Prevention (CDC), Fort Collins, CO, USA [106]. The virus was passaged in C6/36 cells cultured in L15 medium supplemented with 3% fetal bovine serum (FBS), 50 μg/mL penicillin-streptomycin, and 2 mM L-glutamine by infecting the cells at a multiplicity of infection (MOI) of 0.01. Fresh medium was replaced at 7 days post-infection. At 12–14 days post-infection, virus-containing supernatant and infected cells were harvested to prepare the infectious blood meal.

### Mosquito rearing

*Ae*. *aegypti* strain Chetumal was originally collected from Yucatan Peninsula, Mexico [107]. Adult mosquitoes were fed on raisins and water, with uninfected blood meals to stimulate oogenesis, and were maintained at 28°C, 80% relative humidity with 12–12 h light-dark periods. Male mosquitoes (20–25 males) were placed in one-pint cartons with 200–250 female mosquitoes to maintain the colony.

### Mosquito infection with DENV

Mosquitoes were orally exposed to a DENV-infectious blood meal using an artificial membrane feeder as described previously [108] but raisins and water were only removed at 24 and 4 hours prior to blood feeding. For the uninfected control group, we mixed uninfected C6/36 cells suspended in cell culture medium with blood meal to maintain as similar a metabolic input to the DENV-infected group as possible. Mosquitoes were allowed to feed for 45–60 mins. Blood-engorged mosquitoes were reared up to 11 days and fed on sucrose and water.

### Sample preparation for metabolomics analysis

#### Mosquito dissection

On days 3, 7 and 11 pbm, midguts were dissected from 200 uninfected and 300 DENV-infected mosquitoes. Individual midguts were ground in 100 μl phosphate buffered saline (PBS). Two microliters of each individual DENV-exposed midgut (n = 799) homogenates were collected in a separate tube for qRT-PCR analysis. The rest of the homogenate was immediately frozen on dry ice and stored at -80°C for metabolite extraction.

#### Quantitative RT-PCR detection of infected mosquitoes

To determine the presence of DENV RNA in the midgut of mosquitoes following an infectious blood meal, 2μL of midgut homogenate was heated at 95°C for 5 mins and chilled on ice prior to performing a one-step quantitative RT-PCR (qRT-PCR) reaction without RNA extraction. Presence of viral RNA was quantified using Brilliant III Ultra-Fast SYBR qRT-PCR Master Mix (Aligent Technologies, CA) and with the primer pairs for DENV2 RNA as described previously [109]. Quantitative RT-PCR was performed using a iQ5 real-time PCR detection system using the following cycles: RT at 50°C, 20 min; 95°C, 5 min for RT inactivation and 40 cycles of 95°C, 5 s and 60°C, 1 min. The results were analyzed with the iQ5 optical system software version 2.0 (Bio-Rad, CA).

#### Metabolite extraction

Two equal pools of 75–100 midgut homogenates for DENV-infected and mock infected treatment groups were subjected to metabolic extraction. Internal standards (10 μg Alanine-Leucine-Alanine-Leucine and 10 μg Ceramide d17:1, Avanti, AL) were added to the pools for normalization of the variation introduced during tissue extraction. To extract metabolites, one volume of methanol and chloroform and 0.01% butylated hydroxyl toluene were added to an equal volume of homogenous tissue in PBS [110]. Samples were vortexed thoroughly, prior to separation of the aqueous phase from the organic phase by centrifugation at 1,250 x g for 10 minutes. To prevent degradation of metabolites, samples were processed on ice or at 4°C throughout the process. Metabolites from aqueous and organic phases were dried separately by speed-vacuum centrifugation and were stored at -80°C for further metabolomics analysis.

### Liquid chromatography—Mass spectrometry (LC-MS) profiling analysis of metabolites

Two technical replicates of each pool of midguts were analyzed by a LTQ Orbitrap XL instrument (Thermo Scientific, Waltham, MA). It was coupled to an Agilent 1100 series LC (Agilent Technologies, Santa Clara, CA) equipped with a refrigerated well plate auto sampler and binary pumping device.

For polar metabolite analysis, reverse phase liquid chromatography was used to analyze the samples. An Atlantis T3 column (Waters Corp., Milford, MA) with 1.0 x 150 mm, 5.0 μm dimensions was used for the separation. Solvent A consisted of water + 0.1% formic acid. Solvent B consisted of acetonitrile + 0.1% formic acid. The flow rate was 140 μL/minute. A volume of 5 μL was loaded onto the column. The gradient was as follows: time 0 minutes, 0% B; time 1 minutes, 0% B; time 41 minutes, 95% B; time 46 minutes, 95% B; time 50 minutes, 0% B; time 60 minutes 0% B. The LC-MS analysis was run twice, using positive and negative polarity electrospray ionization (ESI). Data were acquired using data dependent scanning mode. FTMS resolution of 60,000 with a mass range of 50–1100 was used for full scan analysis and the FTMS was used for MS/MS data acquisition with a resolution of 7500 and collision induced dissociation (CID) mode.

For non-polar metabolite analysis, reverse phase liquid chromatography was also used to analyze the samples. An Xterra C18 column (Waters Corp., Milford, MA) with 2.1 x 150 mm, 5.0 μm dimensions was used for the separation. Solvent A consisted of water + 10mM ammonium acetate + 0.1% formic acid. Solvent B consisted of acetonitrile/isopropyl alcohol (50/ 50 v/v) + 10mM ammonium acetate + 0.1% formic acid. The flow rate was 300 μL/minute. A volume of 10 μL was loaded onto the column. The gradient was as follows: time 0 minutes, 35% B; time 10 minutes, 80% B; time 20 minutes, 100% B; time 32 minutes, 100% B; time 35 minutes, 35% B; time 40 minutes 35% B. The LC-MS analysis was run twice, using positive and negative polarity in ESI. For MS/MS experiments, data were acquired using a data dependent scanning mode. FTMS resolution of 60,000 with a mass range of 100–1200 was used for full scan analysis and the FTMS was used for MS/MS data acquisition with a resolution of 7500 and higher-energy collisional dissociation (HCD) mode. The top five most intense ions were acquired with a minimum signal of 500, isolation width of 2, normalized collision energy of 35 eV, default charge state of 1, activation Q of 0.250, and an activation time of 30.0. The acquired data were evaluated with Thermo XCalibur software (version 2.1.0).

### MS data processing and statistical methods

Raw data were converted to.mzXML in centroid form using msConvert [111]. Downstream analyses were conducted in the open source R program [112]. The xcms package was used with the centWave [112–114] algorithm and a Gaussian fit for peak-picking, and the OBI-Warp method for retention time correction and alignment [115]. Parameters used are given in the supplement material. Features that had retention times outside of limits determined by the gradient of solutions used were not included in further analysis (2–33 minutes for nonpolar modes and 0–47 minutes for polar modes). Intensities for technical replicates were averaged to provide a single value for each biological replicate and then normalized using median ratio scaling described by Wang, et al. [116]. One was added to intensity values prior to transformation to log_2_ because of the presence of zeroes. Statistical analysis was conducted in the R package limma [117], which fits linear models to the data using an empirical Bayes approach, allowing for information across all features to be used to develop inference about individual features [118]. P-values were based on a moderated t-statistic with a Bayesian adjusted denominator and adjusted for false discovery rate [119]. Each mode (negative / positive + polar / nonpolar) was processed and analyzed separately. Features were designated as significantly different when the absolute log_2_ fold change was greater than or equal to one, and the adjusted p-value was less than 0.05. Where results from all modes are included in one table or graph, the significance is based on the analysis in single modes alone. Putative molecular names of features were identified by searching mass per charge ratio (*m/z*) against the Human Metabolome Database (HMDB), LIPID Metabolites and Pathways Strategy (LIPID MAPS) and Metabolite and Tandem MS Database (Metlin) [118]. [M+H]^+^, [M+Na]^+^ and [M+NH_4_]^+^ adducts were accounted for in the neutral mass calculation in the positive ionization mode nonpolar phase data and [M+H]^+^, [M+Na]^+^ for the polar phase data. [M-H]^-^ was the only adduct accounted for in the calculation of negative ionization mode data. The identifiable molecules with mass accuracy of <6 ppm error was further classified into metabolic classes. Features with MS/MS data were further validated by searching against the NIST MS/MS (2014) (Agilent Technologies) and LipidBlast (2012) [120] libraries using MS PepSearch (2013) [121] and manually inspected using NIST MS Search (v2.0) [122].

### Generating long double-stranded RNA for mosquito gene expression knockdown by RNAi

Long double-stranded RNA (dsRNA) was reversed transcribed and amplified from *Ae aegypti* mosquito total RNA. Briefly, total RNA was extracted from a whole mosquito homogenized in TRIzol reagent (Ambion). Primers were designed to amplify a region of ~ 500 bp of the gene of interest and a T7 promotor sequence was added to the 5’ end of both forward and reverse primers (S2 Table). Reverse transcription reaction (RT) and polymerase chain reaction (PCR) were performed in two-steps using SuperScript III Reverse Transcriptase (Invitrogen) and *Taq* polymerase (NEB) respectively. PCR products were purified using the GeneJET PCR Purification kit (Thermo Scientific) and subjected to *in vitro* transcription using the MEGAscript T7 kit (Invitrogen) according to the manufacturer’s protocol. The reactions were incubated at 37°C for 12 h. The transcribed products were then heated to 75°C for 5 minutes and cooled down at room temperature for 4 h to allow dsRNA to anneal. The products were then treated with DNase to get rid of the DNA template and purified by phenol-chloroform followed by ethanol precipitation. The purified dsRNAs were stored at -80°C for future use.

### Double stranded RNA knockdown of genes in Aag2 cells

*Aedes aegypti* (Aag2) cells were cultured in Schneider’s insect medium (Sigma-Aldrich) supplemented with 2 mM L-glutamine, 1% non-essential amino acids, and 10% FBS. To perform dsRNA knockdown in Aag2 cells, the cells were seeded in a 48-well plate at 50,000 cells/well. Twenty four hours later, the cells were transfected with 260 ng of dsRNA mixed with TransIT-2020 Reagent (Mirus) following the manufacturer’s protocol. New medium with 2% FBS was replaced at 6 hours post transfection. Cell viability assays were performed at 2 days post transfection using the CellTiter-Glo Luminescent Cell Viability Assay (Promega).

### DENV infection of the RNAi-knockdown cells

Cells were infected with DENV at 48 h post dsRNA transfection. To prepare virus inoculum, DENV-containing cell culture supernatant was mixed with 1x PBS supplemented with 0.5mM MgCl_2_, 1.2mM CaCl_2_ and 1% FBS to make a final concentration of virus at a MOI of 0.3. Medium from Aag2 cells was removed and replaced with 0.3 mL of DENV inoculum per well. The cells were allowed to absorb viruses at room temperature for 1 h. Then, the inoculum was removed and replaced with fresh Minimum Essential Medium (MEM; Gibco) supplemented with 1% non-essential amino acids, 2mM L-glutamine and 2% FBS. Quantification of infectious DENV was carried out by plaque assay on BHK cells [6]. Absolute quantification of intracellular DENV genome replication was performed by qRT-PCR as previously described in ‘quantitative RT-PCR detection of infected mosquitoes’ section above. DENV RNA standard was produced from *in vitro* transcribed DENV genome as previously described in [123].

### Quantifying mosquito gene expression upon dsRNA knockdown

Cells were collected in 200 μl TRIzol reagent at two days post dsRNA transfection. RNA extraction was performed following the manufacture’s protocol. Total RNA (500 ng) was subjected to RT using random primers (Invitrogen). The cDNA was quantified by quantitative PCR (qPCR) using PowerUp SYBR Green Master Mix (Applied biosystems). Primer sequences designed to quantify DEGS and actin (internal control) genes are shown in S3 Table. ΔΔCt method was used for calculating percent gene expression of the target gene compared to the GFP knockdown control.

### 4HPR inhibitor studies in Aag2 cells

Aag2 cells pre-treated with 3.75 μM of N-(4-hydroxyphenyl) retinamide (4HPR; Sigma-Aldrich) dissolved in DMSO or DMSO only as a vehicle control. The final concentration of DMSO was at 1% in Schneider media supplemented with 2% FBS. At 24 h post treatment, cells were infected with DENV at MOI of 0.3. Following absorption, cells were overlaid with MEM containing 2% FBS and fresh 4HPR or DMSO. Virus-containing supernatant was harvested at 24 hpi and titrated by plaque assay. Cell viability assays were performed with 4HPR or DMSO treatment without DENV infection using the CellTiter-Glo Luminescent Cell Viability Assay (Promega).

### Quantification of SPs in Aag2 cells by multiple reaction monitoring (MRM)

SPs were extracted from Aag2 cells following the protocol published by [124]. Briefly, cells were trypsinized and washed twice in PBS. Equal cell numbers between treated and control samples were collected. Cer/Sph mixture I Internal standard (Avanti Polar Lipids) was premixed with chloroform at a final concentration of 2 nM. Metabolic extraction was performed as described. Metabolites were dried using nitrogen gas.

An Agilent 1200 Rapid Resolution liquid chromatography (LC) system coupled to an Agilent 6460 series QQQ mass spectrometer (MS) was used to analyze SPs in each sample according to Merrill et al., 2005 with some modifications [124]. A Waters Xbridge C18 2.1mm x 100mm, 3.5 μm column was used for all LC separations (Waters Corp. Milford, MA). The buffers were (A) methanol/water/formic acid (74/25/1 v/v) + 5mM ammonium formate and (B) methanol/formic acid (99/1 v/v) + 5mM ammonium formate for all analyses. All extracted, dried samples were reconstituted in 200 μL of 80/20 buffer A/B just prior to analysis and 10 μL was injected for each analysis. All data were analyzed with Agilent MassHunter Quantitative Analysis (Version B.06.00). The detailed analysis of different SPs is shown in supplemental materials and methods section (S4–S6 Tables).

## Supporting information

S1 Fig *Ae*. *aegypti* sample preparation for LC-MS analysis. Flow chart shows timeline, numbers of samples and procedures used for mosquito rearing, infection, sample collection and sample processing.
(TIF)

S2 Fig 4HPR treatment resulted in increased accumulations of both Cer and DHCer and inhibited DENV replication but did not alter the Cer/DHCer ratios. (A) and (B) Aag2 cells were pre-treated with 3.75 μM of 4HPR or DMSO, a vehicle control. At 24 h post treatment, cells were infected with DENV at MOI of 0.3. Fresh medium with 4HPR or DMSO was replaced at 1 h after absorption. At 24 hpi, (A) cell culture supernatant was harvested and analyzed for infectious viral particle release by plaque assay and (B) total RNA was extracted from infected cells to determine viral genome equivqlents by qRT-PCR. (C) Cell viability test was performed on cells treated with various concentrations of 4HPR. DMSO was used as vehicle only control. (D -G) MRM profiling of SPs in 4HPR or DMSO treated Aag2 cells (N = 3). The cells were treated with 3.75 μM of 4HPR or DMSO. Medium with fresh 4HPR or DMSO was replaced at 24 h after treatment (to mimic the 4HPR treatment of DENV-infected cells) and cells were harvested at 24 h post medium changed. SPs that were profiled are as follow: (D, lower panel) Cer(d18:1/xx:x) and DHCer(d18:0/xx:x) with 18-carbon long chain sphingoid bases (E, lower panel) Cer(d16:1/xx:x) and DHCer(d16:0/xx:x) with 16- carbon long chain sphingoid bases, (F) sphingosine (d18:1), sphingosine-1-phosphate (d18:1-P) and sphinganine (d18:0), (G) sphingomyelin. (D and E, upper panel) showed Cer/DHCer ratios of the Cer and DHCer species with same fatty acyl chain length. These ratios demonstrated that Cer/DHCer ratios were not altered by 4HPR treatment. Student’s t-test was applied to compare the differences in infectious virus release (A), virus genome replication (B) or abundance of SPs (C-F) upon 4HPR treatment to DMSO control. *, p < 0.05; **, p < 0.01.
(TIF)

S3 Fig MRM profiling of additional SPs in Aag2 cells after DEGS-KD By RNAi. Abundance of (A) sphingosine (d18:1), sphingosine-1-phosphate (d18:1-P) and sphinganine (d18:0) and (B) sphingomyelins upon DEGS-KD was compared to GFP-KD control. Student’s t-test was applied for statistical analysis and none of these metabolites had differential abundance upon DEGS-KD.
(TIF)

S4 Fig MRM profiling of SPs in Aag2 cells during DENV infection. DENV infected (MOI of 3) or mock infected Aag2 cells were harvested at 24 hpi and processed for SP profiling by MRM (N = 3). (A, lower panel) Cer(d18:1/xx:x) and DHCer(d18:0/xx:x) with 18-carbon long chain sphingoid bases, and (B, lower panel) Cer(d16:1/xx:x) and DHCer(16:0/xx:x) with 16-carbon long chain sphingoid bases. Cer/DHCer ratios of the species that has the same fatty acyl chain length (e.g. Cer(d18:1/16:0) and DHCer(d18:0/16:0)) were calculated and shown in (A) and (B) upper panels. (C) Sphingosine (d18:1), sphingosine -1-phosphate (d18:1-P) and sphinganine (d18:0), (D) sphingomyelin, Student’s t-test was applied for statistical analysis. *, *p* < 0.05, **, p < 0.01.
(TIF)

S5 Fig Comparative analysis of fatty acyls in mosquito midguts following DENV infection. Average abundance of fatty acyl molecule in DENV infected midguts was compared with uninfected midguts and represented as log_2_ fold change. Each row shows a different fatty acyl molecule, grouped based on the classification of molecular structure. Columns represent 3, 7, and 11 day pbm. Log_2_ fold changes that are zero represent the changes that were not significantly different in DENV infected versus uninfected tissues. Log_2_ fold changes shown in dark red or dark blue represent log_2_ fold changes that are greater than 5 or lower than -5.
(TIF)

S1 Table Select metabolites from mosquito midguts that show differential abundance following DENV infection. Abundance of metabolites detected in DENV-infected and uninfected midguts was compared. Frist tab lists the molecules that were putatively identifiable and second tab lists the molecules were unidentifiable. The following information is provided for each feature: *m/z*_Avg_, average mass to charge ratio detected (averaged between the two biological replicate pools); RT_Avg_, average retention time detected for each metabolite (averaged between the two biological replicate pools); Detection mode, both polar and nonpolar extracts were detected in both negative and positive ionization modes; Name, identification for each lipid; Lipid classes, classification of each lipid according to LIPID MAPS comprehensive classification system [17]; Formula, chemical component of the molecules at their neural mass; Adducts, protonated or deprotonated molecular ions, [M+H]^+^, [M+Na]^+^, [M+NH_4_]^+^ and [M-H]^-^; PPM Error, difference between experimental mass and exact mass; Match ID, accession number for each metabolite; Database, database of the putative identification:LIPID MAPS, Human Metabolome Database or Metlin, Numbers of alternative IDs, other putative names that are isomers of the chosen name at this certain chemical formula; Log_2_ FC, log_2_ fold change of abundance compared between DENV-infected and uninfected samples; p-value, adjusted p-value. MS/MS database, database used for searching tandem MS fragmentation; MSI level, Metabolomics Standard Initiative level of identification [125].
(XLSX)

S2 Table Gene-specific primers for dsRNA. (DOCX)

S3 Table Primers for detecting gene expression levels following RNAi knockdown. (DOCX)

S4 Table Multiple reaction monitoring table for data acquisition of free sphingoid bases and 1-phosphates (according to Merrill et al., 2005 [124]). (DOCX)

S5 Table MRM table for data acquisition of Cer and DHCer (according to Merrill et al. 2005 [124]) and 16 carbon sphingoid-backbone Cer and DHCer (modified from Merrill et al., 2005). (DOCX)

S6 Table MRM table for data acquisition of sphingomyelins (according to Merrill et al., 2005 [124]). (DOCX)

S1 Materials and Methods Analysis of free sphingoid bases and 1-phosphate species, analysis of ceramide (16 and 18 carbon sphingoid-backbone) species, analysis of sphingomyelin species. (DOCX)

## References

[1] Salazar MI, Richardson JH, Sánchez-Vargas I, Olson KE, Beaty BJ. Dengue virus type 2: replication and tropisms in orally infected Aedes aegypti mosquitoes BMC Biology. 2007;7(9). 10.1186/1471-2180-7-9 PMC1797809 17263893

[2] O’GOWER AK. The rate of digestion of human blood by certain species of mosquitoes. Australian Journal of Biological Sciences. 1955;9(1):125–9.

[3] Perera R, Kuhn RJ. Host metabolism and its contribution in Flavivirus biogenesis. In: Arboviruses: Molecular Biology, Evolution and Control in press. Gubler D, Vasilakis N, editors2015, In press.

[4] Junjhon J, Pennington JG, Edwards TJ, Perera R, Lanman J, Kuhn RJ. Ultrastructural characterization and three-dimensional architecture of replication sites in dengue virus-infected mosquito cells. J Virol. 2014;88(9):4687–97. doi: 10.1128/JVI.00118-14 24522909 10.1128/JVI.00118-14 PMC3993787

[5] Perera R, Riley C, Isaac G, Hopf-Jannasch AS, Moore RJ, Weitz KW, et al Dengue virus infection perturbs lipid homeostasis in infected mosquito cells. PLoS Pathog. 2012;8(3):e1002584 doi: 10.1371/journal.ppat.1002584 22457619 10.1371/journal.ppat.1002584 PMC3310792

[6] Heaton NS, Perera R, Berger KL, Khadka S, Lacount DJ, Kuhn RJ, et al Dengue virus nonstructural protein 3 redistributes fatty acid synthase to sites of viral replication and increases cellular fatty acid synthesis. Proc Natl Acad Sci U S A. 2010;107(40):17345–50. doi: 10.1073/pnas.1010811107 20855599 10.1073/pnas.1010811107 PMC2951450

[7] Welsch S, Miller S, Romero-Brey I, Merz A, Bleck CK, Walther P, et al Composition and three-dimensional architecture of the dengue virus replication and assembly sites. Cell Host Microbe. 2009;5(4):365–75. 19380115 10.1016/j.chom.2009.03.007 PMC7103389

[8] Kuhn RJ, Zhang W, Rossmann MG, Pletnev SV, Corver J, Lenches E, et al Structure of dengue virus: implications for flavivirus organization, maturation, and fusion. Cell. 2002;108(5):717–25. 11893341 10.1016/s0092-8674(02)00660-8 PMC4152842

[9] Zaitseva E, Yang ST, Melikov K, Pourmal S, Chernomordik LV. Dengue virus ensures its fusion in late endosomes using compartment-specific lipids. PLoS Pathog. 2010;6(10):e1001131 doi: 10.1371/journal.ppat.1001131 20949067 10.1371/journal.ppat.1001131 PMC2951369

[10] van der Schaar HM, Rust MJ, Chen C, van der Ende-Metselaar H, Wilschut J, Zhuang X, et al Dissecting the cell entry pathway of dengue virus by single-particle tracking in living cells. PLoS Pathog. 2008;4(12):e1000244 doi: 10.1371/journal.ppat.1000244 19096510 10.1371/journal.ppat.1000244 PMC2592694

[11] Paul D, Bartenschlager R. Architecture and biogenesis of plus-strand RNA virus replication factories. World J Virol. 2013;2(2):32–48. doi: 10.5501/wjv.v2.i2.32 24175228 10.5501/wjv.v2.i2.32 PMC3785047

[12] Chatel-Chaix L, Bartenschlager R. Dengue virus- and hepatitis C virus-induced replication and assembly compartments: the enemy inside—caught in the web. J Virol. 2014;88(11):5907–11. doi: 10.1128/JVI.03404-13 24623440 10.1128/JVI.03404-13 PMC4093888

[13] Romero-Brey I, Bartenschlager R. Membranous replication factories induced by plus-strand RNA viruses. Viruses. 2014;6(7):2826–57. doi: 10.3390/v6072826 25054883 10.3390/v6072826 PMC4113795

[14] Girard YA, Popov V, Wen J, Han V, Higgs S. Ultrastructural Study of West Nile Virus Pathogenesis in Culex pipiens quinquefasciatus (Diptera: Culicidae). Journal of Medical Entomology 2005;42(3). 10.1093/jmedent/42.3.429 15962797

[15] Krebs KC, Lan Q. Isolation and expression of a sterol carrier protein-2 gene from the yellow fever mosquito, Aedes aegypti. Insect Mol Biol. 2003;12(1):51–60. 12542635 10.1046/j.1365-2583.2003.00386.x

[16] Clayton RB, Edwards AM, Bloch K. Biosynthesis of cholesterol in an insect, silverfish (Ctenolepisma sp.). Nature. 1962;195:1125–6. 13879846 10.1038/1951125a0

[17] Fahy E, Subramaniam S, Murphy RC, Nishijima M, Raetz CR, Shimizu T, et al Update of the LIPID MAPS comprehensive classification system for lipids. J Lipid Res. 2009;50 Suppl:S9–14. doi: 10.1194/jlr.R800095-JLR200 19098281 10.1194/jlr.R800095-JLR200 PMC2674711

[18] van Meer G, Voelker DR, Feigenson GW. Membrane lipids: where they are and how they behave. Nat Rev Mol Cell Biol. 2008;9(2):112–24. doi: 10.1038/nrm2330 18216768 10.1038/nrm2330 PMC2642958

[19] Dawaliby R, Trubbia C, Delporte C, Noyon C, Ruysschaert JM, Van Antwerpen P, et al Phosphatidylethanolamine Is a Key Regulator of Membrane Fluidity in Eukaryotic Cells. J Biol Chem. 2016;291(7):3658–67. doi: 10.1074/jbc.M115.706523 26663081 10.1074/jbc.M115.706523 PMC4751403

[20] Bohdanowicz M, Grinstein S. Role of phospholipids in endocytosis, phagocytosis, and macropinocytosis. Physiol Rev. 2013;93(1):69–106. doi: 10.1152/physrev.00002.2012 23303906 10.1152/physrev.00002.2012

[21] Divecha N, Irvine RF. Phospholipid signaling. Cell. 1995;80(2):269–78. 7834746 10.1016/0092-8674(95)90409-3

[22] Guan XL, Cestra G, Shui G, Kuhrs A, Schittenhelm RB, Hafen E, et al Biochemical membrane lipidomics during Drosophila development. Dev Cell. 2013;24(1):98–111. doi: 10.1016/j.devcel.2012.11.012 23260625 10.1016/j.devcel.2012.11.012

[23] Sommer U, Molloy J, Viant M, Sinkins S. MTBLS210: Wolbachia modulation of lipid metabolism in Aedes albopictus mosquito cells 2015. http://www.ebi.ac.uk/metabolights/MTBLS210. 10.1128/AEM.00275-16 PMC4959074 26994075

[24] Molloy JC, Sommer U, Viant MR, Sinkins SP. Wolbachia Modulates Lipid Metabolism in Aedes albopictus Mosquito Cells. Appl Environ Microbiol. 2016;82(10):3109–20. doi: 10.1128/AEM.00275-16 26994075 10.1128/AEM.00275-16 PMC4959074

[25] da Rocha Fernandes M, Martins R, Pessoa Costa E, Pacidonio EC, Araujo de Abreu L, da Silva Vaz I Jr., et al The modulation of the symbiont/host interaction between Wolbachia pipientis and Aedes fluviatilis embryos by glycogen metabolism. PLoS One. 2014;9(6):e98966 doi: 10.1371/journal.pone.0098966 24926801 10.1371/journal.pone.0098966 PMC4057193

[26] Caragata EP, Rances E, O’Neill SL, McGraw EA. Competition for amino acids between Wolbachia and the mosquito host, Aedes aegypti. Microb Ecol. 2014;67(1):205–18. doi: 10.1007/s00248-013-0339-4 24337107 10.1007/s00248-013-0339-4

[27] Zhou GL, Flowers M, Friedrich K, Horton J, Pennington J, Wells MA. Metabolic fate of [C-14]-labeled meal protein amino acids in Aedes aegypti mosquitoes. Journal of Insect Physiology. 2004;50(4):337–49. 15081827 10.1016/j.jinsphys.2004.02.003

[28] Atella GC, Shahabuddin M. Differential partitioning of maternal fatty acid and phospholipid in neonate mosquito larvae. J Exp Biol. 2002;205(Pt 23):3623–30. 12409488 10.1242/jeb.205.23.3623

[29] Ziegler R, Van Antwerpen R. Lipid uptake by insect oocytes. Insect Biochem Mol Biol. 2006;36(4):264–72. doi: 10.1016/j.ibmb.2006.01.014 16551540 10.1016/j.ibmb.2006.01.014

[30] Canavoso LE, Frede S, Rubiolo ER. Metabolic pathways for dietary lipids in the midgut of hematophagous Panstrongylus megistus (Hemiptera: Reduviidae). Insect Biochem Mol Biol. 2004;34(8):845–54. doi: 10.1016/j.ibmb.2004.05.008 15262288 10.1016/j.ibmb.2004.05.008

[31] Arrese EL, Canavoso LE, Jouni ZE, Pennington JE, Tsuchida K, Wells MA. Lipid storage and mobilization in insects: current status and future directions. Insect Biochemistry and Molecular Biology. 2001;31(1):7–17. doi: 10.1016/S0965-1748(00)00102-8 11102830 10.1016/s0965-1748(00)00102-8

[32] Sarri E, Sicart A, Lazaro-Dieguez F, Egea G. Phospholipid synthesis participates in the regulation of diacylglycerol required for membrane trafficking at the Golgi complex. J Biol Chem. 2011;286(32):28632–43. doi: 10.1074/jbc.M111.267534 21700701 10.1074/jbc.M111.267534 PMC3151104

[33] Lin YH, Chen YC, Kao TY, Lin YC, Hsu TE, Wu YC, et al Diacylglycerol lipase regulates lifespan and oxidative stress response by inversely modulating TOR signaling in Drosophila and C. elegans. Aging Cell. 2014;13(4):755–64. doi: 10.1111/acel.12232 24889782 10.1111/acel.12232 PMC4116436

[34] Merida I, Avila-Flores A, Merino E. Diacylglycerol kinases: at the hub of cell signalling. Biochem J. 2008;409(1):1–18. doi: 10.1042/BJ20071040 18062770 10.1042/BJ20071040

[35] Pennington JE, Nussenzveig RH, Van Heusden MC. Lipid transfer from insect fat body to lipophorin: comparison between a mosquito triacylglycerol-rich lipophorin and a sphinx moth diacylglycerol-rich lipophorin. J Lipid Res. 1996;37(5):1144–52. . 8725165

[36] Cheon HM, Shin SW, Bian G, Park JH, Raikhel AS. Regulation of lipid metabolism genes, lipid carrier protein lipophorin, and its receptor during immune challenge in the mosquito Aedes aegypti. J Biol Chem. 2006;281(13):8426–35. doi: 10.1074/jbc.M510957200 16449228 10.1074/jbc.M510957200

[37] Martin-Acebes MA, Merino-Ramos T, Blazquez AB, Casas J, Escribano-Romero E, Sobrino F, et al The composition of West Nile virus lipid envelope unveils a role of sphingolipid metabolism in flavivirus biogenesis. J Virol. 2014;88(20):12041–54. doi: 10.1128/JVI.02061-14 25122799 10.1128/JVI.02061-14 PMC4178726

[38] Hannun YA, Obeid LM. Principles of bioactive lipid signalling: lessons from sphingolipids. Nat Rev Mol Cell Biol. 2008;9(2):139–50. doi: 10.1038/nrm2329 18216770 10.1038/nrm2329

[39] Jan JT, Chatterjee S, Griffin DE. Sindbis virus entry into cells triggers apoptosis by activating sphingomyelinase, leading to the release of ceramide. J Virol. 2000;74(14):6425–32. 10864654 10.1128/jvi.74.14.6425-6432.2000 PMC112150

[40] Schneider-Schaulies J, Schneider-Schaulies S. Viral infections and sphingolipids. Handb Exp Pharmacol. 2013;(216):321–40. doi: 10.1007/978-3-7091-1511-4_16 23563664 10.1007/978-3-7091-1511-4_16

[41] Merrill AH Jr. Sphingolipid and glycosphingolipid metabolic pathways in the era of sphingolipidomics. Chem Rev. 2011;111(10):6387–422. doi: 10.1021/cr2002917 21942574 10.1021/cr2002917 PMC3191729

[42] Hanada K. Serine palmitoyltransferase, a key enzyme of sphingolipid metabolism. Biochim Biophys Acta. 2003;1632(1–3):16–30. 12782147 10.1016/s1388-1981(03)00059-3

[43] Chen Y, Liu Y, Sullards MC, Merrill AH Jr. An introduction to sphingolipid metabolism and analysis by new technologies. Neuromolecular Med. 2010;12(4):306–19. doi: 10.1007/s12017-010-8132-8 20680704 10.1007/s12017-010-8132-8 PMC2982954

[44] Maceyka M, Milstien S, Spiegel S. Sphingosine kinases, sphingosine-1-phosphate and sphingolipidomics. Prostaglandins Other Lipid Mediat. 2005;77(1–4):15–22. doi: 10.1016/j.prostaglandins.2004.09.010 16099387 10.1016/j.prostaglandins.2004.09.010

[45] Maceyka M, Sankala H, Hait NC, Le Stunff H, Liu H, Toman R, et al SphK1 and SphK2, sphingosine kinase isoenzymes with opposing functions in sphingolipid metabolism. Journal of Biological Chemistry. 2005;280(44):37118–29. doi: 10.1074/jbc.M502207200 16118219 10.1074/jbc.M502207200

[46] Vacaru AM, van den Dikkenberg J, Ternes P, Holthuis JC. Ceramide phosphoethanolamine biosynthesis in Drosophila is mediated by a unique ethanolamine phosphotransferase in the Golgi lumen. J Biol Chem. 2013;288(16):11520–30. doi: 10.1074/jbc.M113.460972 23449981 10.1074/jbc.M113.460972 PMC3630839

[47] Liu YY, Hill RA, Li YT. Ceramide glycosylation catalyzed by glucosylceramide synthase and cancer drug resistance. Adv Cancer Res. 2013;117:59–89. doi: 10.1016/B978-0-12-394274-6.00003-0 23290777 10.1016/B978-0-12-394274-6.00003-0 PMC4051614

[48] Rodriguez-Cuenca S, Barbarroja N, Vidal-Puig A. Dihydroceramide desaturase 1, the gatekeeper of ceramide induced lipotoxicity. Biochim Biophys Acta. 2015;1851(1):40–50. doi: 10.1016/j.bbalip.2014.09.021 25283058 10.1016/j.bbalip.2014.09.021

[49] Michel C, van Echten-Deckert G, Rother J, Sandhoff K, Wang E, Merrill AH Jr. Characterization of ceramide synthesis. A dihydroceramide desaturase introduces the 4,5-trans-double bond of sphingosine at the level of dihydroceramide. J Biol Chem. 1997;272(36):22432–7. 9312549 10.1074/jbc.272.36.22432

[50] Carocci M, Hinshaw SM, Rodgers MA, Villareal VA, Burri DJ, Pilankatta R, et al The bioactive lipid 4-hydroxyphenyl retinamide inhibits flavivirus replication. Antimicrob Agents Chemother. 2015;59(1):85–95. doi: 10.1128/AAC.04177-14 25313218 10.1128/AAC.04177-14 PMC4291433

[51] Delia D, Aiello A, Formelli F, Fontanella E, Costa A, Miyashita T, et al Regulation of apoptosis induced by the retinoid N-(4-hydroxyphenyl) retinamide and effect of deregulated bcl-2. Blood. 1995;85(2):359–67. 7811993

[52] Horvath TD, Dagan S, Lorenzi PL, Hawke DH, Scaraffia PY. Positional stable isotope tracer analysis reveals carbon routes during ammonia metabolism of Aedes aegypti mosquitoes. FASEB J. 2017 doi: 10.1096/fj.201700657R 28970248 10.1096/fj.201700657R PMC5731135

[53] Arrese EL, Soulages JL. Insect fat body: energy, metabolism, and regulation. Annu Rev Entomol. 2010;55:207–25. doi: 10.1146/annurev-ento-112408-085356 19725772 10.1146/annurev-ento-112408-085356 PMC3075550

[54] Canavoso LE, Jouni ZE, Karnas KJ, Pennington JE, Wells MA. Fat metabolism in insects. Annu Rev Nutr. 2001;21:23–46. doi: 10.1146/annurev.nutr.21.1.23 11375428 10.1146/annurev.nutr.21.1.23

[55] Stanley DW, Miller JS. Eicosanoid actions in insect cellular immune functions. Entomologia Experimentalis Et Applicata. 2006;119(1):1–13. doi: 10.1111/j.1570-7458.2006.00406.x

[56] Brezinski ME, Serhan CN. Selective incorporation of (15S)-hydroxyeicosatetraenoic acid in phosphatidylinositol of human neutrophils: agonist-induced deacylation and transformation of stored hydroxyeicosanoids. Proc Natl Acad Sci U S A. 1990;87(16):6248–52. 2117277 10.1073/pnas.87.16.6248 PMC54510

[57] Zhang G, Kodani S, Hammock BD. Stabilized epoxygenated fatty acids regulate inflammation, pain, angiogenesis and cancer. Prog Lipid Res. 2014;53:108–23. doi: 10.1016/j.plipres.2013.11.003 24345640 10.1016/j.plipres.2013.11.003 PMC3914417

[58] Funk CD, Powell WS. Metabolism of linoleic acid by prostaglandin endoperoxide synthase from adult and fetal blood vessels. Biochim Biophys Acta. 1983;754(1):57–71. 6414520 10.1016/0005-2760(83)90082-6

[59] Tortoriello G, Rhodes BP, Takacs SM, Stuart JM, Basnet A, Raboune S, et al Targeted Lipidomics in Drosophila melanogaster Identifies Novel 2-Monoacylglycerols and N-acyl Amides. Plos One. 2013;8(7). doi: 10.1371/journal.pone.0067865 23874457 10.1371/journal.pone.0067865 PMC3708943

[60] Gregersen N, Kolvraa S, Rasmussen K, Christensen E, Brandt NJ, Ebbesen F, et al Biochemical studies in a patient with defects in the metabolism of acyl-CoA and sarcosine: another possible case of glutaric aciduria type II. J Inherit Metab Dis. 1980;3(3):67–72. 6158623 10.1007/BF02312527

[61] Koves TR, Ussher JR, Noland RC, Slentz D, Mosedale M, Ilkayeva O, et al Mitochondrial overload and incomplete fatty acid oxidation contribute to skeletal muscle insulin resistance. Cell Metab. 2008;7(1):45–56. doi: 10.1016/j.cmet.2007.10.013 18177724 10.1016/j.cmet.2007.10.013

[62] Lasser NL, Clayton RB. The intracellular distribution of sterols in Eurycotis floridana and its possible relation to subcellular membrane structures. J Lipid Res. 1966;7(3):413–21. 5929357

[63] Lasser NL, Edwards AM, Clayton RB. Distribution and dynamic state of sterols and steroids in the tissues of an insect, the roach Eurycotis floridana. J Lipid Res. 1966;7(3):403–12. 5929356

[64] Rees HH. Ecdysteroid Biosynthesis and Inactivation in Relation to Function. European Journal of Entomology. 1995;92(1):9–39.

[65] Clayton RB. The Utilization of Sterols by Insects. J Lipid Res. 1964;5:3–19. 14173327

[66] Clark AJ, Bloch K. Conversion of ergosterol to 22-de-hydrocholesterol in Blattella germanica. J Biol Chem. 1959;234:2589–94. 13810424

[67] Lee CJ, Lin HR, Liao CL, Lin YL. Cholesterol effectively blocks entry of flavivirus. J Virol. 2008;82(13):6470–80. doi: 10.1128/JVI.00117-08 18448543 10.1128/JVI.00117-08 PMC2447114

[68] Carro AC, Damonte EB. Requirement of cholesterol in the viral envelope for dengue virus infection. Virus Res. 2013;174(1–2):78–87. doi: 10.1016/j.virusres.2013.03.005 23517753 10.1016/j.virusres.2013.03.005

[69] Mackenzie JM, Khromykh AA, Parton RG. Cholesterol manipulation by West Nile virus perturbs the cellular immune response. Cell Host Microbe. 2007;2(4):229–39. doi: 10.1016/j.chom.2007.09.003 18005741 10.1016/j.chom.2007.09.003

[70] Nchoutmboube JA, Viktorova EG, Scott AJ, Ford LA, Pei Z, Watkins PA, et al Increased long chain acyl-Coa synthetase activity and fatty acid import is linked to membrane synthesis for development of picornavirus replication organelles. PLoS Pathog. 2013;9(6):e1003401 doi: 10.1371/journal.ppat.1003401 23762027 10.1371/journal.ppat.1003401 PMC3675155

[71] Heaton NS, Randall G. Dengue virus-induced autophagy regulates lipid metabolism. Cell Host Microbe. 2010;8(5):422–32. doi: 10.1016/j.chom.2010.10.006 21075353 10.1016/j.chom.2010.10.006 PMC3026642

[72] Mathews CK, Holde KEV, Appling DR, Anthony-Cahill SJ. Biochemistry. 4th ed Toronto: Pearson Canada; 2013.

[73] Ye YH, Woolfit M, Rances E, O’Neill SL, McGraw EA. Wolbachia-associated bacterial protection in the mosquito Aedes aegypti. PLoS Negl Trop Dis. 2013;7(8):e2362 doi: 10.1371/journal.pntd.0002362 23951381 10.1371/journal.pntd.0002362 PMC3738474

[74] Scaraffia PY, Zhang Q, Thorson K, Wysocki VH, Miesfeld RL. Differential ammonia metabolism in Aedes aegypti fat body and midgut tissues. J Insect Physiol. 2010;56(9):1040–9. doi: 10.1016/j.jinsphys.2010.02.016 20206632 10.1016/j.jinsphys.2010.02.016 PMC2910787

[75] Sanders HR, Evans AM, Ross LS, Gill SS. Blood meal induces global changes in midgut gene expression in the disease vector, Aedes aegypti. Insect Biochem Mol Biol. 2003;33(11):1105–22. 14563362 10.1016/s0965-1748(03)00124-3

[76] Ziegler R, Ibrahim MM. Formation of lipid reserves in fat body and eggs of the yellow fever mosquito, Aedes aegypti. Journal of Insect Physiology. 2001;47(6):623–7. doi: 10.1016/S0022-1910(00)00158-X 11249951 10.1016/s0022-1910(00)00158-x

[77] Price DP, Nagarajan V, Churbanov A, Houde P, Milligan B, Drake LL, et al The fat body transcriptomes of the yellow fever mosquito Aedes aegypti, pre- and post- blood meal. PLoS One. 2011;6(7):e22573 doi: 10.1371/journal.pone.0022573 21818341 10.1371/journal.pone.0022573 PMC3144915

[78] Koller CN, Raikhel AS. Initiation of Vitellogenin Uptake and Protein-Synthesis in the Mosquito (Aedes-Aegypti) Ovary in Response to a Blood Meal. Journal of Insect Physiology. 1991;37(9):703–11. doi: 10.1016/0022-1910(91)90048-5

[79] Giraldo-Calderon GI, Emrich SJ, MacCallum RM, Maslen G, Dialynas E, Topalis P, et al VectorBase: an updated bioinformatics resource for invertebrate vectors and other organisms related with human diseases. Nucleic Acids Res. 2015;43(Database issue):D707–13. doi: 10.1093/nar/gku1117 25510499 10.1093/nar/gku1117 PMC4383932

[80] Brown DA, London E. Structure and function of sphingolipid- and cholesterol-rich membrane rafts. J Biol Chem. 2000;275(23):17221–4. doi: 10.1074/jbc.R000005200 10770957 10.1074/jbc.R000005200

[81] Scheitz CJ, Guo Y, Early AM, Harshman LG, Clark AG. Heritability and inter-population differences in lipid profiles of Drosophila melanogaster. PLoS One. 2013;8(8):e72726 doi: 10.1371/journal.pone.0072726 24013349 10.1371/journal.pone.0072726 PMC3754969

[82] Kraut R. Roles of sphingolipids in Drosophila development and disease. J Neurochem. 2011;116(5):764–78. doi: 10.1111/j.1471-4159.2010.07022.x 21214556 10.1111/j.1471-4159.2010.07022.x

[83] Fyrst H, Saba JD. Sphingosine-1-phosphate lyase in development and disease: sphingolipid metabolism takes flight. Biochim Biophys Acta. 2008;1781(9):448–58. Epub 2008/06/19. doi: 10.1016/j.bbalip.2008.05.005 18558101 10.1016/j.bbalip.2008.05.005 PMC2749932

[84] Khalil SM, Rompp A, Pretzel J, Becker K, Spengler B. Phospholipid Topography of Whole-Body Sections of the Anopheles stephensi Mosquito, Characterized by High-Resolution Atmospheric-Pressure Scanning Microprobe Matrix-Assisted Laser Desorption/Ionization Mass Spectrometry Imaging. Anal Chem. 2015;87(22):11309–16. doi: 10.1021/acs.analchem.5b02781 26491885 10.1021/acs.analchem.5b02781

[85] Yang TK, Means E, Anderson LE, Jenkin HM. Sphingophospholipids of species of Aedes and Culex mosquito cells cultivated in suspension culture from logarithmic and stationary phases of growth. Lipids. 1974;9(12):1009–13. 4444421 10.1007/BF02533827

[86] Aktepe TE, Pham H, Mackenzie JM. Differential utilisation of ceramide during replication of the flaviviruses West Nile and dengue virus. Virology. 2015;484:241–50. doi: 10.1016/j.virol.2015.06.015 26122470 10.1016/j.virol.2015.06.015

[87] Bremer J. The role of carnitine in intracellular metabolism. J Clin Chem Clin Biochem. 1990;28(5):297–301. . 2199593

[88] Lopaschuk GD, Ussher JR, Folmes CD, Jaswal JS, Stanley WC. Myocardial fatty acid metabolism in health and disease. Physiol Rev. 2010;90(1):207–58. doi: 10.1152/physrev.00015.2009 20086077 10.1152/physrev.00015.2009

[89] Schulz H. Oxidation of fatty acids in eukaryotes In Biochemistry of Lipids, Lipoproteins and Membranes. 5th ed Vance DE, Vance J, editors. Amsterdam: Elsevier; 2008.

[90] Rutkowsky JM, Knotts TA, Ono-Moore KD, McCoin CS, Huang S, Schneider D, et al Acylcarnitines activate proinflammatory signaling pathways. Am J Physiol Endocrinol Metab. 2014;306(12):E1378–87. doi: 10.1152/ajpendo.00656.2013 24760988 10.1152/ajpendo.00656.2013 PMC4059985

[91] Knabb MT, Saffitz JE, Corr PB, Sobel BE. The dependence of electrophysiological derangements on accumulation of endogenous long-chain acyl carnitine in hypoxic neonatal rat myocytes. Circ Res. 1986;58(2):230–40. 3948341 10.1161/01.res.58.2.230

[92] Bakermans AJ, van Weeghel M, Denis S, Nicolay K, Prompers JJ, Houten SM. Carnitine supplementation attenuates myocardial lipid accumulation in long-chain acyl-CoA dehydrogenase knockout mice. J Inherit Metab Dis. 2013;36(6):973–81. doi: 10.1007/s10545-013-9604-4 23563854 10.1007/s10545-013-9604-4

[93] Kler RS, Jackson S, Bartlett K, Bindoff LA, Eaton S, Pourfarzam M, et al Quantitation of acyl-CoA and acylcarnitine esters accumulated during abnormal mitochondrial fatty acid oxidation. J Biol Chem. 1991;266(34):22932–8. 1744086

[94] El-Bacha T, Midlej V, Pereira da Silva AP, Silva da Costa L, Benchimol M, Galina A, et al Mitochondrial and bioenergetic dysfunction in human hepatic cells infected with dengue 2 virus. Biochim Biophys Acta. 2007;1772(10):1158–66. doi: 10.1016/j.bbadis.2007.08.003 17964123 10.1016/j.bbadis.2007.08.003

[95] Fontaine KA, Sanchez EL, Camarda R, Lagunoff M. Dengue virus induces and requires glycolysis for optimal replication. J Virol. 2015;89(4):2358–66. doi: 10.1128/JVI.02309-14 25505078 10.1128/JVI.02309-14 PMC4338897

[96] Fullekrug J, Ehehalt R, Poppelreuther M. Outlook: membrane junctions enable the metabolic trapping of fatty acids by intracellular acyl-CoA synthetases. Front Physiol. 2012;3:401 doi: 10.3389/fphys.2012.00401 23087649 10.3389/fphys.2012.00401 PMC3467455

[97] Eyster KM. The membrane and lipids as integral participants in signal transduction: lipid signal transduction for the non-lipid biochemist. Adv Physiol Educ. 2007;31(1):5–16. doi: 10.1152/advan.00088.2006 17327576 10.1152/advan.00088.2006

[98] Fernandis AZ, Wenk MR. Membrane lipids as signaling molecules. Curr Opin Lipidol. 2007;18(2):121–8. 17353659 10.1097/MOL.0b013e328082e4d5

[99] Caragata EP, Rances E, Hedges LM, Gofton AW, Johnson KN, O’Neill SL, et al Dietary cholesterol modulates pathogen blocking by Wolbachia. PLoS Pathog. 2013;9(6):e1003459 doi: 10.1371/journal.ppat.1003459 23825950 10.1371/journal.ppat.1003459 PMC3694857

[100] Clayton RB, Hinkle PC, Smith DA, Edwards AM. The Intestinal Absorption of Cholesterol, Its Esters and Some Related Sterols and Analogues in the Roac, Eurycotis Floridana. Comp Biochem Physiol. 1964;11:333–50. 14167530 10.1016/0010-406x(64)90001-5

[101] Matsumoto K. Dispensable nature of phosphatidylglycerol in Escherichia coli: dual roles of anionic phospholipids. Mol Microbiol. 2001;39(6):1427–33. 11260460 10.1046/j.1365-2958.2001.02320.x

[102] Ishii I, Fukushima N, Ye X, Chun J. Lysophospholipid receptors: signaling and biology. Annu Rev Biochem. 2004;73:321–54. doi: 10.1146/annurev.biochem.73.011303.073731 15189145 10.1146/annurev.biochem.73.011303.073731

[103] Mishima K, Nakajima M, Ogihara T. Effects of lysophospholipids on membrane order of phosphatidylcholine. Colloids and Surfaces B-Biointerfaces. 2004;33(3–4):185–9. doi: 10.1016/j.colsurfb.2003.10.004

[104] Brackney DE, Scott JC, Sagawa F, Woodward JE, Miller NA, Schilkey FD, et al C6/36 Aedes albopictus cells have a dysfunctional antiviral RNA interference response. PLoS Negl Trop Dis. 2010;4(10):e856 doi: 10.1371/journal.pntd.0000856 21049065 10.1371/journal.pntd.0000856 PMC2964293

[105] Stollar V, Thomas VL. An agent in the Aedes aegypti cell line (Peleg) which causes fusion of Aedes albopictus cells. Virology. 1975;64(2):367–77. 806166 10.1016/0042-6822(75)90113-0

[106] Deubel V, Kinney RM, Trent DW. Nucleotide sequence and deduced amino acid sequence of the structural proteins of dengue type 2 virus, Jamaica genotype. Virology. 1986;155(2):365–77. 3024394 10.1016/0042-6822(86)90200-x

[107] Bennett KE, Olson KE, Munoz Mde L, Fernandez-Salas I, Farfan-Ale JA, Higgs S, et al Variation in vector competence for dengue 2 virus among 24 collections of Aedes aegypti from Mexico and the United States. Am J Trop Med Hyg. 2002;67(1):85–92. 12363070 10.4269/ajtmh.2002.67.85

[108] Richardson J, Molina-Cruz A, Salazar MI, Black Wt. Quantitative analysis of dengue-2 virus RNA during the extrinsic incubation period in individual Aedes aegypti. Am J Trop Med Hyg. 2006;74(1):132–41. 16407358

[109] Laue T, Emmerich P, Schmitz H. Detection of dengue virus RNA in patients after primary or secondary dengue infection by using the TaqMan automated amplification system. J Clin Microbiol. 1999;37(8):2543–7. 10405398 10.1128/jcm.37.8.2543-2547.1999 PMC85278

[110] Bligh EG, Dyer WJ. A rapid method of total lipid extraction and purification. Can J Biochem Physiol. 1959;37(8):911–7. doi: 10.1139/o59-099 13671378 10.1139/o59-099

[111] Team RC. R: A language and environment for statistical computing Vienna, Austria 2015 http://www.R-project.org/.

[112] Benton HP, Want EJ, Ebbels TM. Correction of mass calibration gaps in liquid chromatography-mass spectrometry metabolomics data. Bioinformatics. 2010;26(19):2488–9. Epub 2010/07/31. doi: 10.1093/bioinformatics/btq441 20671148 10.1093/bioinformatics/btq441

[113] Smith CA, Want EJ, O’Maille G, Abagyan R, Siuzdak G. XCMS: processing mass spectrometry data for metabolite profiling using nonlinear peak alignment, matching, and identification. Anal Chem. 2006;78(3):779–87. doi: 10.1021/ac051437y 16448051 10.1021/ac051437y

[114] Tautenhahn R, Bottcher C, Neumann S. Highly sensitive feature detection for high resolution LC/MS. BMC Bioinformatics. 2008;9:504 doi: 10.1186/1471-2105-9-504 19040729 10.1186/1471-2105-9-504 PMC2639432

[115] Prince JT, Marcotte EM. Chromatographic alignment of ESI-LC-MS proteomics data sets by ordered bijective interpolated warping. Anal Chem. 2006;78(17):6140–52. doi: 10.1021/ac0605344 16944896 10.1021/ac0605344

[116] Wang W, Zhou H, Lin H, Roy S, Shaler TA, Hill LR, et al Quantification of proteins and metabolites by mass spectrometry without isotopic labeling or spiked standards. Anal Chem. 2003;75(18):4818–26. 14674459 10.1021/ac026468x

[117] Ritchie ME, Phipson B, Wu D, Hu Y, Law CW, Shi W, et al limma powers differential expression analyses for RNA-sequencing and microarray studies. Nucleic Acids Res. 2015;43(7):e47 doi: 10.1093/nar/gkv007 25605792 10.1093/nar/gkv007 PMC4402510

[118] Smyth GK. Linear models and empirical bayes methods for assessing differential expression in microarray experiments. Stat Appl Genet Mol Biol. 2004;3:Article3. doi: 10.2202/1544-6115.1027 16646809 10.2202/1544-6115.1027

[119] Benjamini Y, Hochberg Y. Controlling the false discovery rate: a practical and powerful approach to multiple testing. Journal of the Royal Statistical Society. 1995:289–300.

[120] Kind T, Liu KH, Lee DY, DeFelice B, Meissen JK, Fiehn O. LipidBlast in silico tandem mass spectrometry database for lipid identification. Nat Methods. 2013;10(8):755–8. doi: 10.1038/nmeth.2551 23817071 10.1038/nmeth.2551 PMC3731409

[121] MS PepSearch 2013. http://chemdata.nist.gov/dokuwiki/doku.php?id=peptidew:mspepsearch#restrictions_and_disclaimers.

[122] Mass Spectrum Interpreter Ver. 3. http://chemdata.nist.gov/dokuwiki/doku.php?id=chemdata:interpreter

[123] Butrapet S, Kinney RM, Huang CY. Determining genetic stabilities of chimeric dengue vaccine candidates based on dengue 2 PDK-53 virus by sequencing and quantitative TaqMAMA. J Virol Methods. 2006;131(1):1–9. doi: 10.1016/j.jviromet.2005.06.019 16087248 10.1016/j.jviromet.2005.06.019

[124] Merrill AH Jr., Sullards MC, Allegood JC, Kelly S, Wang E. Sphingolipidomics: high-throughput, structure-specific, and quantitative analysis of sphingolipids by liquid chromatography tandem mass spectrometry. Methods. 2005;36(2):207–24. doi: 10.1016/j.ymeth.2005.01.009 15894491 10.1016/j.ymeth.2005.01.009

[125] Sumner LW, Amberg A, Barrett D, Beale MH, Beger R, Daykin CA, et al Proposed minimum reporting standards for chemical analysis Chemical Analysis Working Group (CAWG) Metabolomics Standards Initiative (MSI). Metabolomics. 2007;3(3):211–21. doi: 10.1007/s11306-007-0082-2 24039616 10.1007/s11306-007-0082-2 PMC3772505

