# Supplementary figures and images for: Dynamic remodeling of lipids coincides with dengue virus replication in the midgut of Aedes aegypti mosquitoes
Source: PLoS Pathog. 2018 Feb 15;14(2):e1006853. doi: 10.1371/journal.ppat.1006853 (PMC5814098; doi:10.1371/journal.ppat.1006853)

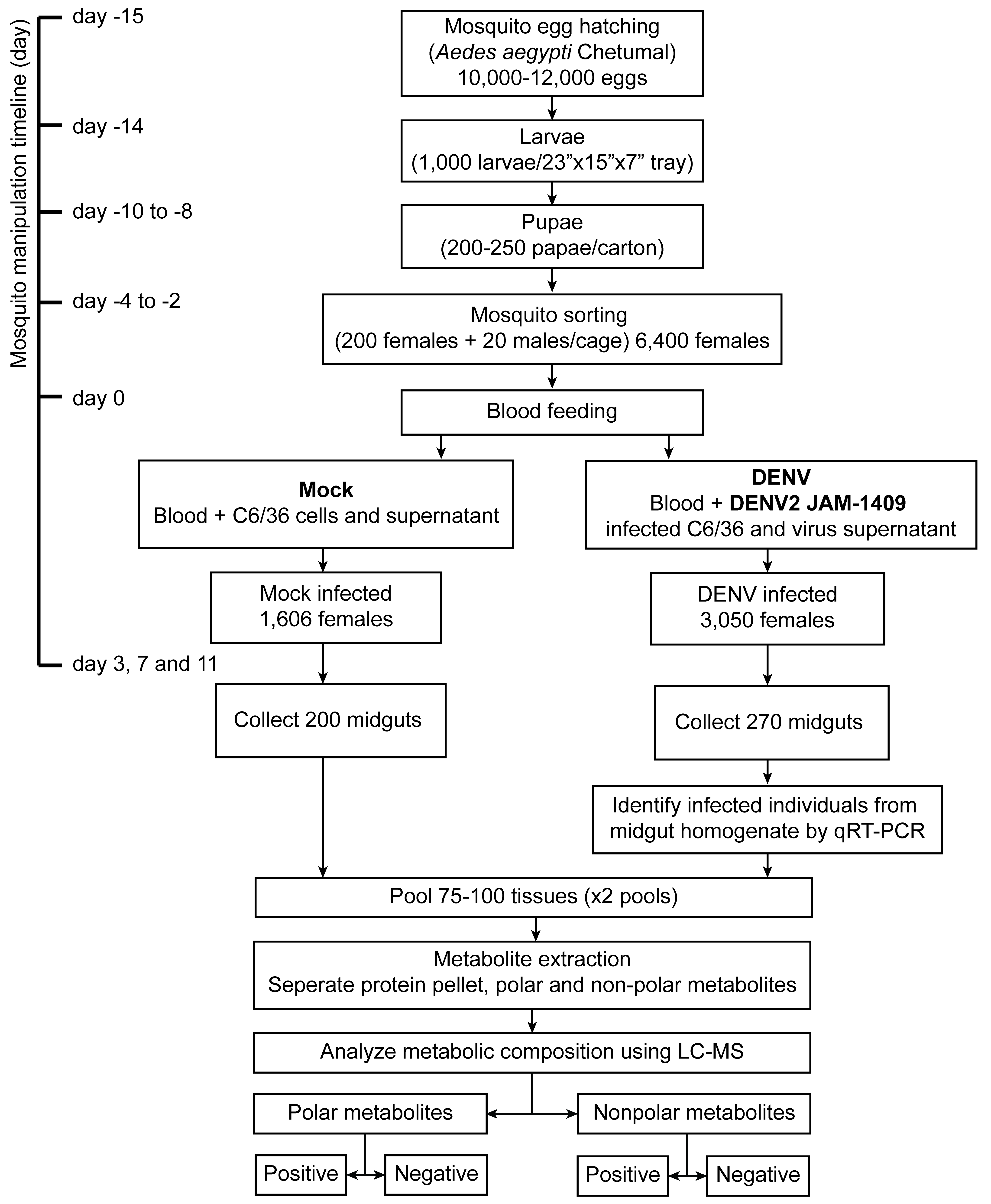

Supplement: S1 Fig — Flow chart shows timeline, numbers of samples and procedures used for mosquito rearing, infection, sample collection and sample processing. (TIF) [file ppat.1006853.s001.tif]

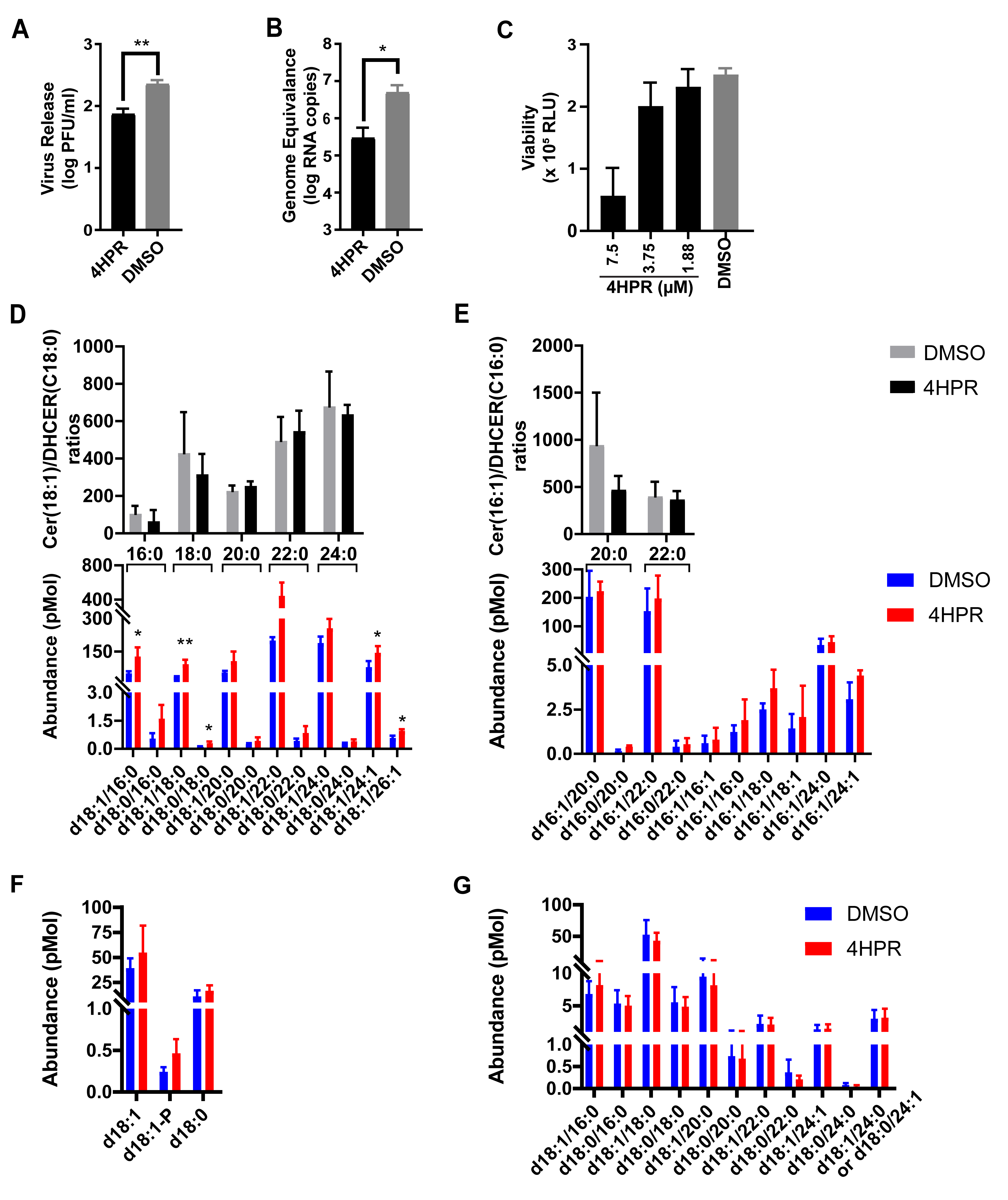

Supplement: S2 Fig — (A) and (B) Aag2 cells were pre-treated with 3.75 μM of 4HPR or DMSO, a vehicle control. At 24 h post treatment, cells were infected with DENV at MOI of 0.3. Fresh medium with 4HPR or DMSO was replaced at 1 h after absorption. At 24 hpi, (A) cell culture supernatant was harvested and analyzed for infectious viral particle release by plaque assay and (B) total RNA was extracted from infected cells to determine viral genome equivqlents by qRT-PCR. (C) Cell viability test was performed on cells treated with various concentrations of 4HPR. DMSO was used as vehicle only control. (D -G) MRM profiling of SPs in 4HPR or DMSO treated Aag2 cells (N = 3). The cells were treated with 3.75 μM of 4HPR or DMSO. Medium with fresh 4HPR or DMSO was replaced at 24 h after treatment (to mimic the 4HPR treatment of DENV-infected cells) and cells were harvested at 24 h post medium changed. SPs that were profiled are as follow: (D, lower panel) Cer(d18:1/xx:x) and DHCer(d18:0/xx:x) with 18-carbon long chain sphingoid bases (E, lower panel) Cer(d16:1/xx:x) and DHCer(d16:0/xx:x) with 16- carbon long chain sphingoid bases, (F) sphingosine (d18:1), sphingosine-1-phosphate (d18:1-P) and sphinganine (d18:0), (G) sphingomyelin. (D and E, upper panel) showed Cer/DHCer ratios of the Cer and DHCer species with same fatty acyl chain length. These ratios demonstrated that Cer/DHCer ratios were not altered by 4HPR treatment. Student’s t-test was applied to compare the differences in infectious virus release (A), virus genome replication (B) or abundance of SPs (C-F) upon 4HPR treatment to DMSO control. *, p < 0.05; **, p < 0.01. (TIF) [file ppat.1006853.s002.tif]

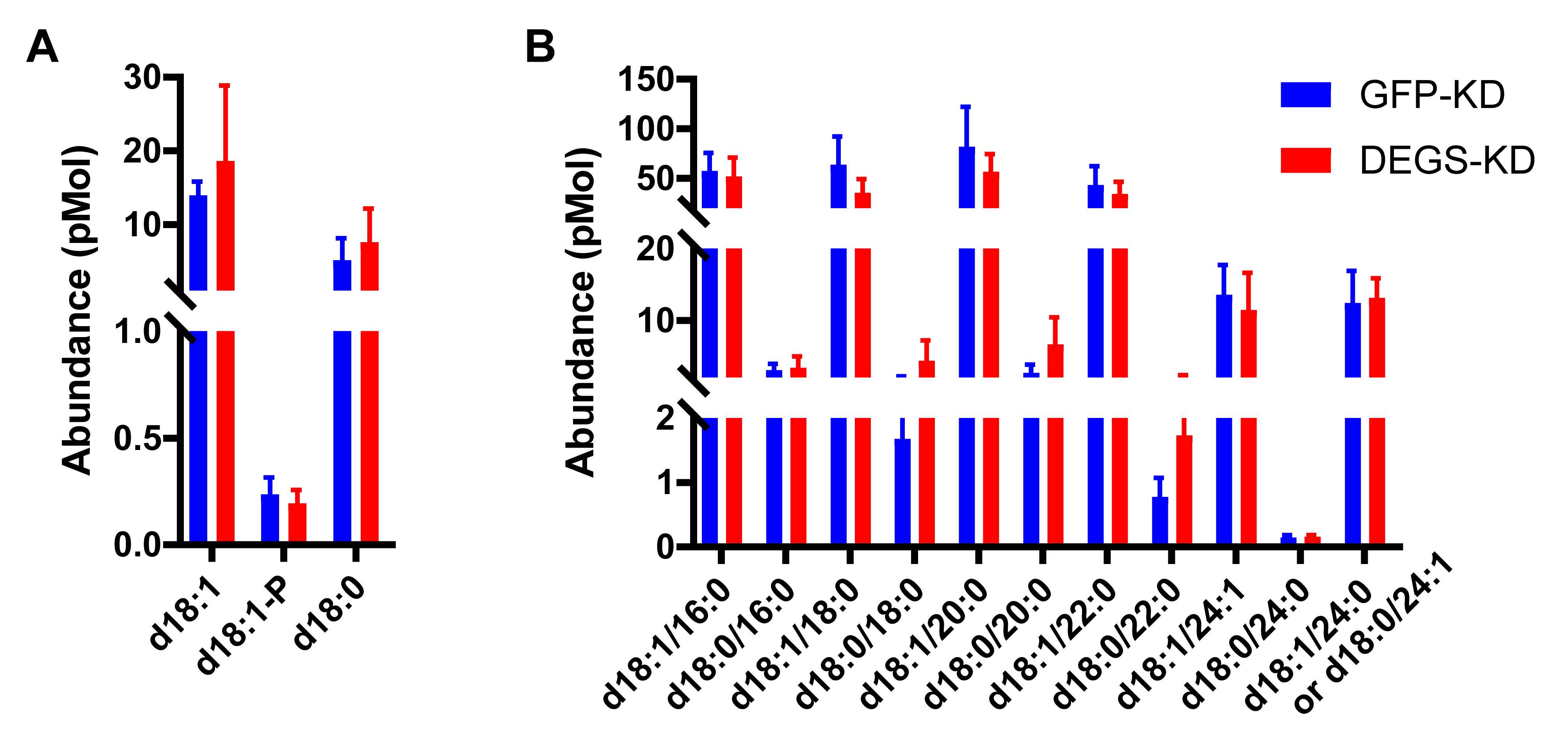

Supplement: S3 Fig — Abundance of (A) sphingosine (d18:1), sphingosine-1-phosphate (d18:1-P) and sphinganine (d18:0) and (B) sphingomyelins upon DEGS-KD was compared to GFP-KD control. Student’s t-test was applied for statistical analysis and none of these metabolites had differential abundance upon DEGS-KD. (TIF) [file ppat.1006853.s003.tif]

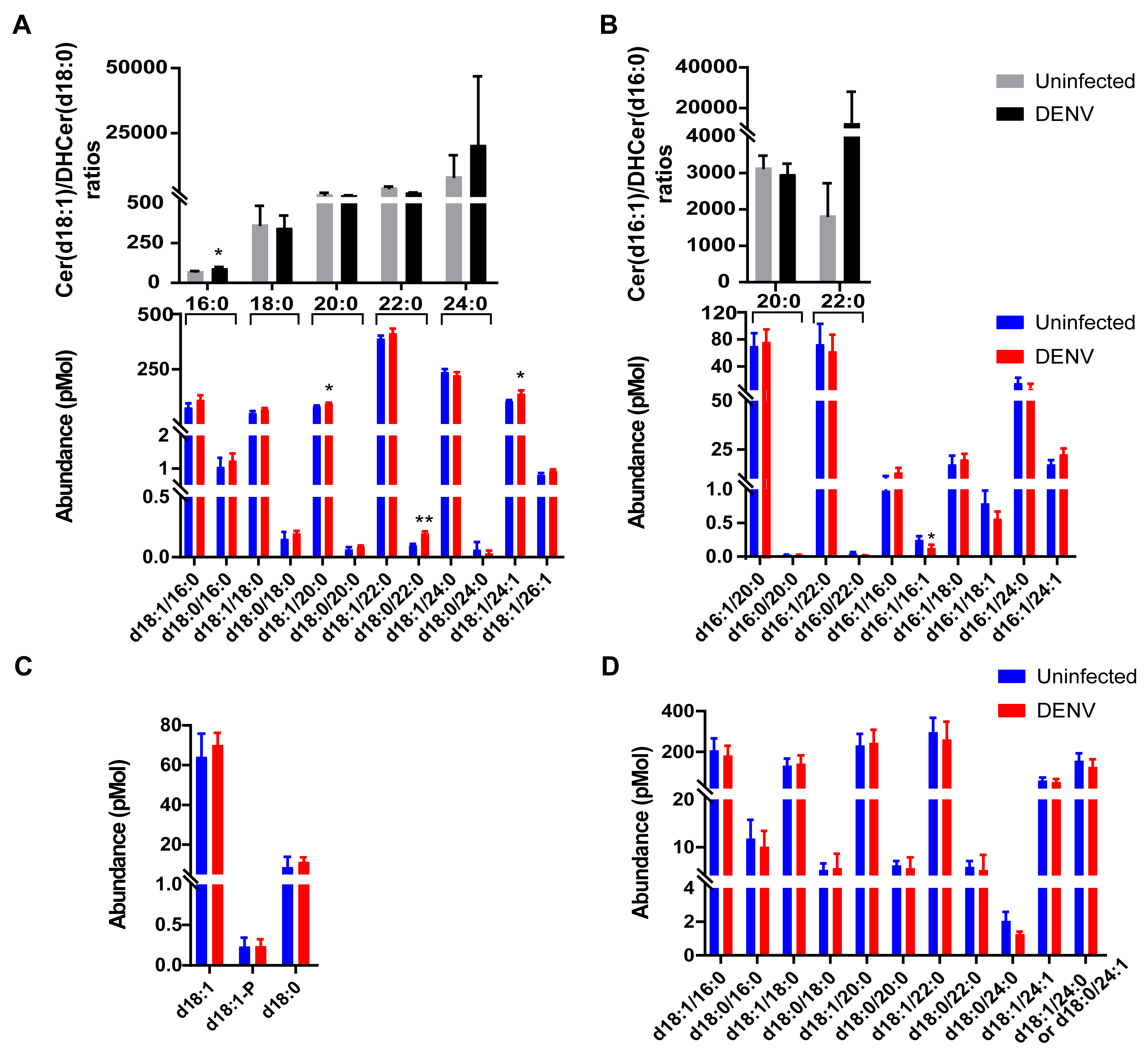

Supplement: S4 Fig — DENV infected (MOI of 3) or mock infected Aag2 cells were harvested at 24 hpi and processed for SP profiling by MRM (N = 3). (A, lower panel) Cer(d18:1/xx:x) and DHCer(d18:0/xx:x) with 18-carbon long chain sphingoid bases, and (B, lower panel) Cer(d16:1/xx:x) and DHCer(16:0/xx:x) with 16-carbon long chain sphingoid bases. Cer/DHCer ratios of the species that has the same fatty acyl chain length (e.g. Cer(d18:1/16:0) and DHCer(d18:0/16:0)) were calculated and shown in (A) and (B) upper panels. (C) Sphingosine (d18:1), sphingosine -1-phosphate (d18:1-P) and sphinganine (d18:0), (D) sphingomyelin, Student’s t-test was applied for statistical analysis. *, p < 0.05, **, p < 0.01. (TIF) [file ppat.1006853.s004.tif]

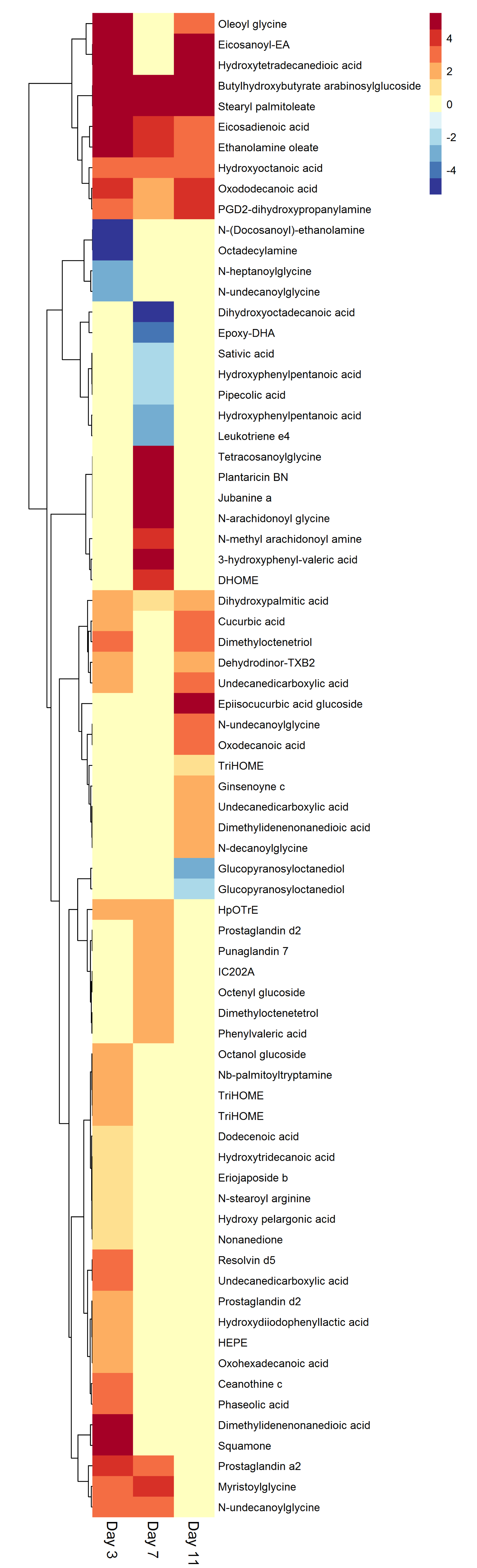

Supplement: S5 Fig — Average abundance of fatty acyl molecule in DENV infected midguts was compared with uninfected midguts and represented as log2 fold change. Each row shows a different fatty acyl molecule, grouped based on the classification of molecular structure. Columns represent 3, 7, and 11 day pbm. Log2 fold changes that are zero represent the changes that were not significantly different in DENV infected versus uninfected tissues. Log2 fold changes shown in dark red or dark blue represent log2 fold changes that are greater than 5 or lower than -5. (TIF) [file ppat.1006853.s005.tif]
