# Supplementary material for: Dynamic remodeling of lipids coincides with dengue virus replication in the midgut of Aedes aegypti mosquitoes
Source: PLoS Pathog. 2018 Feb 15;14(2):e1006853. doi: 10.1371/journal.ppat.1006853 (PMC5814098; doi:10.1371/journal.ppat.1006853)
Supplement: S2 Table — (DOCX) [file ppat.1006853.s007.docx]

**S2 Table. Gene-specific primers for dsRNA**

| Primer name | Sequence (5’ to 3’)* | Target gene |
| --- | --- | --- |
| DEGS-dsRNA_F | ATACAGATTTGCCAACGCTG | *Ae aegypti* DEGS  (VB: AAEL013047) |
| DEGS-dsRNA_R | GCTTCGGATTTACGATCAGC | *Ae aegypti* DEGS  (VB: AAEL013047) |
| GFP-dsRNA_F | GACCACATGAAGCAGCACGA | eGFP |
| GFP-dsRNA_R | CGCTTCTCGTTGGGGTCTTT | eGFP |
| DENV-dsRNA_F | ACGGAGAACCACACATGATCG | DENV serotype 2  (GB: U87411.1) |
| DENV-dsRNA_R | CTCCTGAAACCCCTTCCACAA | DENV serotype 2  (GB: U87411.1) |

* T7 promotor sequence (5’ GAATTAATACGACTCACTATAGGGAGA 3’) was added to the 5’ end of all primers to make dsRNA

VB, VectorBase accession number; GB, GenBank accession number
