# Supplementary material for: Dynamic remodeling of lipids coincides with dengue virus replication in the midgut of Aedes aegypti mosquitoes
Source: PLoS Pathog. 2018 Feb 15;14(2):e1006853. doi: 10.1371/journal.ppat.1006853 (PMC5814098; doi:10.1371/journal.ppat.1006853)
Supplement: S3 Table — (DOCX) [file ppat.1006853.s008.docx]

**S3 Table. Primers for detecting gene expression levels following RNAi knockdown**

| Primer name | Sequence (5’ to 3’) |
| --- | --- |
| DEGS-qPCR_F | ATACAGATTTGCCAACGCTG |
| DEGS-qPCR_R | GCTTCGGATTTACGATCAGC |
| Actin-qPCR_F | GAATGTGCAAGGCCGGATTC |
| Actin-qPCR_R | GCTCGATCGGGTACTTCAGG |
