# Supplementary material for: Dynamic remodeling of lipids coincides with dengue virus replication in the midgut of Aedes aegypti mosquitoes
Source: PLoS Pathog. 2018 Feb 15;14(2):e1006853. doi: 10.1371/journal.ppat.1006853 (PMC5814098; doi:10.1371/journal.ppat.1006853)
Supplement: S4 Table — (DOCX) [file ppat.1006853.s009.docx]

**S4 Table. Multiple reaction monitoring table for data acquisition of free sphingoid bases and 1-phosphates (according to Merrill et al., 2005 [124])**

| **Compound** | **Precursor (*m/z*)** | **Product (*m/z*)** | **Collision Energy (V)** |
| --- | --- | --- | --- |
| d17:0 | 288.3 | 252.3 | 12 |
| d18:1 | 300.3 | 264.3 | 12 |
| d18:0 | 302.3 | 266.3 | 12 |
| t18:0 | 318.3 | 282.3 | 12 |
| d20:1 | 328.4 | 292.3 | 12 |
| d20:0 | 330.3 | 294.3 | 12 |
| d17:1-P | 366.4 | 250.3 | 12 |
| d17:0-P | 368.4 | 252.3 | 12 |
| d18:1-P | 380.4 | 264.3 | 12 |
| d18:0-P | 382.4 | 266.3 | 12 |
| d20:1-P | 408.4 | 292.3 | 12 |
| d20:0-P | 410.4 | 294.3 | 12 |
| d17:1 | 286.3 | 250.3 | 12 |
| Dimethylsphingosine | 328.4 | 310.3 | 15 |
| Trimethylsphingosine | 342.4 | 60.2 | 20 |
