# Supplementary material for: Dynamic remodeling of lipids coincides with dengue virus replication in the midgut of Aedes aegypti mosquitoes
Source: PLoS Pathog. 2018 Feb 15;14(2):e1006853. doi: 10.1371/journal.ppat.1006853 (PMC5814098; doi:10.1371/journal.ppat.1006853)
Supplement: S5 Table — (DOCX) [file ppat.1006853.s010.docx]

**S5 Table. MRM table for data acquisition of Cer and DHCer (according to Merrill et al. 2005 [124]) and 16 carbon sphingoid-backbone Cer and DHCer (modified from Merrill et al., 2005)**

| **Compound** | **Precursor (*m/z*)** | **Product (*m/z*)** | **Collision Energy (V)** |
| --- | --- | --- | --- |
| d18:1/16:0 | 538.7 | 264.3 | 20 |
| d18:0/16:0 | 540.7 | 266.3 | 20 |
| d18:1/18:0 | 566.7 | 264.3 | 20 |
| d18:0/18:0 | 568.7 | 266.3 | 20 |
| d18:1/20:0 | 594.7 | 264.3 | 20 |
| d18:0/20:0 | 596.7 | 266.3 | 20 |
| d18:1/22:0 | 622.8 | 264.3 | 20 |
| d18:0/22:0 | 624.8 | 266.3 | 20 |
| d18:1/24:1 | 648.9 | 264.3 | 20 |
| d18:0/24:1 | 650.9 | 266.3 | 20 |
| d18:1/24:0 | 650.9 | 264.3 | 20 |
| d18:0/24:0 | 652.9 | 266.3 | 20 |
| d18:1/25:0 | 664.7 | 264.3 | 20 |
| d18:1/26:1 | 676.9 | 264.3 | 20 |
| d18:0/26:1 | 678.9 | 266.3 | 20 |
| d18:0/26:0 | 678.9 | 264.3 | 20 |
| d18:0/26:0 | 680.9 | 266.3 | 20 |
| d18:0/12:0 | 482.6 | 264.3 | 20 |
| d16:0/26:0 | 652.7 | 238.3 | 20 |
| d16:0/26:1 | 650.6 | 238.3 | 20 |
| d16:1/26:0 | 650.6 | 236.3 | 20 |
| d16:1/26:1 | 648.6 | 236.3 | 20 |
| d16:0/24:0 | 624.6 | 238.3 | 20 |
| d16:0/24:1 | 622.6 | 238.3 | 20 |
| d16:1/24:0 | 622.6 | 236.3 | 20 |
| d16:1/24:1 | 620.6 | 236.3 | 20 |
| d16:0/22:0 | 596.6 | 238.3 | 20 |
| d16:1/22:0 | 594.5 | 236.3 | 20 |
| d16:0/20:0 | 568.6 | 238.3 | 20 |
| d16:1/20:0 | 566.5 | 236.3 | 20 |
| d16:0/18:0 | 540.5 | 238.3 | 20 |
| d16:0/18:1 | 538.5 | 238.3 | 20 |
| d16:1/18:0 | 538.5 | 236.3 | 20 |
| d16:0/18:2 | 536.5 | 238.3 | 20 |
| d16:1/18:1 | 536.5 | 236.3 | 20 |
| d16:1/18:2 | 534.5 | 236.3 | 20 |
| d16:0/16:0 | 512.5 | 238.3 | 20 |
| d16:0/16:1 | 510.5 | 238.3 | 20 |
| d16:1/16:0 | 510.5 | 236.3 | 20 |
| d16:1/16:1 | 508.5 | 236.3 | 20 |
| d18:0/12:0 | 482.6 | 264.3 | 20 |
| d18:1/25:0 | 664.7 | 264.3 | 20 |
