# Supplementary material for: Dynamic remodeling of lipids coincides with dengue virus replication in the midgut of Aedes aegypti mosquitoes
Source: PLoS Pathog. 2018 Feb 15;14(2):e1006853. doi: 10.1371/journal.ppat.1006853 (PMC5814098; doi:10.1371/journal.ppat.1006853)
Supplement: S6 Table — (DOCX) [file ppat.1006853.s011.docx]

**S6 Table. MRM table for data acquisition of sphingomyelins (according to Merrill et al., 2005 [124])**

| **Compound** | **Precursor (*m/z*)** | **Product (*m/z*)** | **Collision Energy (V)** |
| --- | --- | --- | --- |
| d18:1/16:0 | 703.8 | 184.4 | 20 |
| d18:0/16:0 | 705.8 | 184.4 | 20 |
| d18:1/18:0 | 731.8 | 184.4 | 20 |
| d18:0/18:0 | 733.8 | 184.4 | 20 |
| d18:1/20:0 | 759.8 | 184.4 | 20 |
| d18:0/20:0 | 761.8 | 184.4 | 20 |
| d18:1/22:0 | 787.9 | 184.4 | 20 |
| d18:0/22:0 | 789.9 | 184.4 | 20 |
| d18:1/24:1 | 813.9 | 184.4 | 20 |
| d18:0/24:1 | 815.9 | 184.4 | 20 |
| d18:1/24:0 | 815.9 | 184.4 | 20 |
| d18:0/24:0 | 817.9 | 184.4 | 20 |
| d18:1/26:1 | 841.9 | 184.4 | 20 |
| d18:0/26:1 | 843.9 | 184.4 | 20 |
| d18:1/26:0 | 843.9 | 184.4 | 20 |
| d18:0/26:0 | 845.9 | 184.4 | 20 |
| d18:1/12:0 | 647.7 | 184.4 | 20 |
