# Supplementary material for: Dynamic remodeling of lipids coincides with dengue virus replication in the midgut of Aedes aegypti mosquitoes
Source: PLoS Pathog. 2018 Feb 15;14(2):e1006853. doi: 10.1371/journal.ppat.1006853 (PMC5814098; doi:10.1371/journal.ppat.1006853)
Supplement: S1 Materials and Methods — (DOCX) [file ppat.1006853.s012.docx]

**S1 Materials and methods**

**Analysis of free sphingoid bases and 1-phosphate species**

The linear LC gradient was as follows: time 0 minutes, 0 % B; time 1 minute, 0 % B; time 10 minutes, 100 % B; time 10.5 minutes, 100 % B; time 11 minutes, 0 % B; time 15 minutes, 0 % B. The flow rate was 0.3 mL/min. MRM was used for MS analysis. The data were acquired in positive ESI mode according to S4 table. The jet stream ESI interface had a gas temperature of 325°C, gas flow rate of 8 L/minute, nebulizer pressure of 45 psi, sheath gas temperature of 250°C, sheath gas flow rate of 7 L/minute, capillary voltage of 4000 V in positive mode, and nozzle voltage of 1000 V. The ΔEMV voltage was 500.

**Analysis of ceramide (16 and 18 carbon sphingoid-backbone) species**

Isocratic conditions of 100% B buffer for 30 minutes were used for the analysis. The flow rate was 0.3 mL/min. Multiple reaction monitoring was used for MS analysis. The data were acquired in positive ESI mode according to S5 table. The jet stream ESI interface had a gas temperature of 325°C, gas flow rate of 8 L/minute, nebulizer pressure of 45 psi, sheath gas temperature of 250°C, sheath gas flow rate of 7 L/minute, capillary voltage of 4000 V in positive mode, and nozzle voltage of 1000 V. The ΔEMV voltage was 400.

**Analysis of sphingomyelin species**

The linear LC gradient was as follows: time 0 minutes, 20 % B; time 1 minute, 20 % B; time 10 minutes, 100 % B; time 20 minutes, 100 % B; time 22 minutes, 20 % B; time 30 minutes, 20 % B. The flow rate was 0.3 mL/min. Multiple reaction monitoring was used for MS analysis. The data were acquired in positive ESI mode according to S6 table. The jet stream ESI interface had a gas temperature of 325°C, gas flow rate of 8 L/minute, nebulizer pressure of 45 psi, sheath gas temperature of 250°C, sheath gas flow rate of 7 L/minute, capillary voltage of 4000 V in positive mode, and nozzle voltage of 1000 V. The ΔEMV voltage was 400.
